# TRIM59/RBPJ positive feedback circuit confers gemcitabine resistance in pancreatic cancer by activating the Notch signaling pathway

**DOI:** 10.1038/s41419-024-07324-y

**Published:** 2024-12-26

**Authors:** Shiyu Chen, Zhiwei He, Kun Cai, Yan Zhang, Hongyan Zhu, Chong Pang, Jiaqi Zhang, Dong Wang, Xundi Xu

**Affiliations:** 1 Department of Hepatobiliary Pancreatic Surgery, South China Hospital, Medical School, Shenzhen University, Shenzhen, 518116 P. R. China; 2 Guangdong Key Laboratory for Biomedical Measurements and Ultrasound Imaging, National-Regional Key Technology Engineering Laboratory for Medical Ultrasound, School of Biomedical Engineering, Shenzhen University Medical School, Shenzhen, 518060 China; 3 Department of Hepatobiliary Surgery, Shenzhen University General Hospital & Shenzhen University Clinical Medical Academy Center, Shenzhen University, Shenzhen, 518000 China; 4 Department of Hepatobiliary Surgery, The Affiliated Hospital of Guizhou Medical University, Guizhou Medical University, Guiyang, 550001 China; 5 Department of Gastrointestinal Surgery, South China Hospital, Medical School, Shenzhen University, Shenzhen, 518116 P. R. China

**Keywords:** Cancer therapeutic resistance, Oncogenes

## Abstract

Pancreatic cancer (PC) is one of the most lethal malignant tumors that lacks effective treatment, and gemcitabine-based chemoresistance occurs frequently. Therefore, new therapeutic strategies for PC are urgently needed. Tripartite motif containing 59 (TRIM59) plays an important role in breast and lung cancer chemoresistance. However, the association between TRIM59 and gemcitabine resistance in PC remains unclear. We identified TRIM59 as an innovative E3 ubiquitin ligase that activated Notch signaling in PC. TRIM59 levels were increased in PC and positively correlated with poor prognosis and gemcitabine resistance in PC patients. TRIM59 facilitated gemcitabine resistance in PC cells in vitro and in vivo. TRIM59 interacted with recombination signal binding protein for immunoglobulin kappa J region (RBPJ) and stabilized it by promoting K63-linked ubiquitination. RBPJ transcriptionally upregulated TRIM59 expression, forming a positive feedback loop with TRIM59. We identified a novel TRIM59 inhibitor, catechin, and confirmed that it sensitized PC cells to gemcitabine. TRIM59 conferred gemcitabine resistance in PC by promoting RBPJ K63-linked ubiquitination, followed by activating Notch signaling. Therefore, our study provides a promising target for gemcitabine sensitization in PC treatment.

## Introduction

Pancreatic cancer (PC) is one of the most lethal malignant tumors [1]. Although advances in diagnostic and therapeutic methods have improved the clinical course of PC, the prognosis remains poor, with a 5-year survival rate of less than 10% [2]. Currently, surgical resection is the only possible cure for PC patients; however, because of the insidious pathogenesis of PC, patients are often diagnosed with advanced disease, and most of them have already developed distant metastasis, thus losing the chance of surgery [3]. Therefore, chemotherapy remains the primary treatment option for inoperable PC patients [4]. Chemotherapeutic agents, such as gemcitabine, play a crucial role in PC treatment [5]. However, as the phenomenon of chemotherapy resistance in PC patients has not been well resolved in clinical practice, it is urgent to find new therapeutic strategies, especially to identify specific therapeutic targets, to further improve the prognosis of PC patients.

The tripartite motif containing (TRIM) family of proteins belongs to a large group of E3 ubiquitin ligases that are composed of a RING-finger domain, a coiled-coil region, one or two B-boxes, and zinc-binding motifs [6]. Currently, the TRIM family proteins are attracting increasing attention for their role in cancer chemoresistance, including lung cancer [7], glioma [8], and colorectal cancer [9]. Recent evidence on tripartite motif containing 59 (TRIM59) indicates that it is involved in the chemoresistance of various cancers. Liu et al. revealed that TRIM59 confers paclitaxel resistance in breast cancer by regulating ubiquitination of p53 [10]. Feng et al. found that TRIM59 enhances the resistance of colorectal cancer cells to bortezomib [11]. Another study revealed that TRIM59 is highly expressed in lung cancer and contributes to gefitinib resistance in EGFR mutant (Mut) lung adenocarcinomas [12]. Our previous studies demonstrated that TRIM31 and TRIM37 confer gemcitabine and fluorouracil resistance in PC by activating the NF-κB and AKT–GSK-3β–β-catenin signaling pathways, respectively [13, 14]. Recently, it is reported that TRIM59 is involved in the modulation of ferroptosis in PC [15]. However, little is known regarding the role of TRIM59 in PC chemoresistance.

The evolutionary conserved Notch signaling pathway is dysregulated in various tumors [16]. During the modulation of Notch signaling, recombination signal binding protein for immunoglobulin kappa J region (RBPJ, also known as CSL) serves as the key transcription factor (TF) that mediates the transcription of downstream target genes, thereby regulating biological properties of tumors [17, 18]. Notch signaling is involved in chemoresistance in many cancers [19–21], including PC [22]. We have previously shown that activation of the Notch pathway leads to enhanced proliferation and motility of PC cells [23]. Nevertheless, the relationship between Notch signaling and TRIM59 in PC chemoresistance remains unclear.

In this study, the innovative E3 ubiquitin ligase TRIM59 was identified, which contributed to an increase in RBPJ protein levels and activation of the Notch signaling pathway. Furthermore, analysis of clinical data from PC patients revealed that increased TRIM59 levels were related to poor prognosis and gemcitabine resistance in PC patients. Moreover, in vitro and in vivo assays verified that TRIM59 facilitated gemcitabine resistance in PC cells. Next, we showed that TRIM59 interacted with RBPJ and facilitated its K63-linked ubiquitination, resulting in RBPJ stabilization. Furthermore, TRIM59 was positively regulated by RBPJ at the transcriptional level, thus forming a positive feedback circuit. Finally, we screened a small-molecule inhibitor of TRIM59, catechin, which is known to sensitize PC cells to gemcitabine. Our current study identified a promising lead compound and sheds new light on the development of sensitizers for PC treatment based on the application of gemcitabine.

## Materials and methods

### Clinical specimens

PC specimens and adjacent non-tumor samples were obtained from the Department of Hepatobiliary Pancreatic Surgery, South China Hospital, Medical School, Shenzhen University, and the Department of Hepatobiliary Surgery, The Affiliated Hospital of Guizhou Medical University. The research protocol was approved by the Ethics Committee of South China Hospital, Medical School, Shenzhen University, and the Ethics Committee of The Affiliated Hospital of Guizhou Medical University. All of the patients provided written informed consent.

### Chemicals and cell culture

Human PC cell lines (MIA PaCa-2, SW1990 and PANC-1) and human embryonic kidney 293 T (HEK-293T) cells were purchased from American Type Culture Collection (Manassas, VA). The cell lines were authenticated via STR profiling and confirmed to be mycoplasma-free. The cells were cultured in Dulbecco’s Modified Eagle’s Medium (Gibco, Waltham, MA) containing 1% streptomycin and penicillin (Gibco) and 10% fetal bovine serum (Gibco) in a humidified incubator (Thermo Fisher Scientific, Waltham, MA) containing 5% CO_2_ at 37 °C. For all of the cellular assays, three independent repeated experiments were performed. Gemcitabine (Selleck), cycloheximide (Sigma), MG132 (Selleck), Y-39983 (MedChemExpress), avatrombopag (Selleck), and catechin (MedChemExpress) were purchased from the indicated suppliers.

### Real-time quantitative polymerase chain reaction (RT-qPCR)

RNA from distinct tissues and cells was extracted using TRIzol reagent (Invitrogen, Carlsbad, CA). PrimeScript RT reagent (TaKaRa, Dalian, Liaoning, China) was used to obtain cDNA. Next, RT-qPCR was performed. All experiments were performed in line with the manufacturer’s protocol, and the 2^−ΔΔCt^ method was used to analyze the results.

### Western blot

PC specimens and cells were lysed using radioimmunoprecipitation assay buffer (Thermo Fisher Scientific) containing a phosphatase inhibitor cocktail and phenylmethanesulfonyl fluoride (Boster Biological Technology; Wuhan, Hubei, China). Proteins were quantified using a bicinchoninic acid assay kit (Biosharp, Hefei, Anhui, China). Sodium dodecyl sulfate-polyacrylamide gel electrophoresis (SDS-PAGE) was performed to separate the proteins, which were then transferred onto PVDF membranes (Millipore, Darmstadt, Germany). The protein bands were detected using specific antibodies (Supplementary Table 1).

### Plasmid transfection and lentivirus infection

Plasmids were obtained from WZ Biosciences Co., Ltd. (Jinan, Shandong, China). TRIM59 overexpression and short hairpin RNA lentiviruses were acquired from GeneChem Co., Ltd. (Shanghai, China). Plasmid transfection and lentivirus infection were performed according to the manufacturer’s protocol.

### Hematoxylin & eosin (H&E) and immunohistochemistry (IHC) staining

The excised samples were fixed with 10% formalin solution, embedded in paraffin, and cut into 4 µm sections. H&E staining was performed on the slices in compliance with the standard histological protocol. For the IHC assay, tissue sections were dewaxed, hydrated, rinsed, antigen retrieved, and blocked successively. Subsequently, primary antibodies including RBPJ, TRIM59, Ki-67, and cleaved caspase 3 were added and incubated at 4 °C overnight. Thereafter, the sections were incubated with the corresponding secondary antibodies for 2 h at 25 °C, followed by DAB staining, rinsing, hematoxylin redyeing, dehydration, and sealing. Five representative visual areas of each slice were assessed and scored by two individuals who were blinded to the study.

### Cell viability assay

Cell Counting Kit-8 (Dojindo Molecular Technologies, Inc., Japan) was used to evaluate cell viability. PC cells (5 × 10^3^/well) were seeded in 96-well plates. Twenty-four hours later, gemcitabine in distinct concentration was added into the wells. Subsequently, CCK-8 solution mixed with the medium (10% final concentration) was used to replace the previous medium. The absorbance was measured at 450 nm using a microplate reader.

### Cell apoptosis assay

Apoptosis in the PC cell lines was examined using a cell apoptosis detection kit (Biosharp). Briefly, PC cells were seeded in 6-well plates, and after the indicated treatment, the cells were collected and stained with 7-AAD and Annexin V according to the manufacturer’s protocol. A FACSCelesta™ multicolor flow cytometer (Becton Dickinson, Franklin Lakes, NJ) was used to assess apoptosis.

### Terminal-deoxynucleotidyl transferase mediated nick end labeling (TUNEL) staining

TUNEL assay was conducted to detect apoptosis in tissues using the One Step TUNEL Apoptosis Assay Kit (Beyotime, Shanghai, China). Briefly, the slices were dewaxed, hydrated, rinsed sequentially, and subjected to TUNEL and DAPI staining. A fluorescence microscope (Olympus, Tokyo, Japan) was used for imaging.

### Colony formation experiment

PC cells were seeded in 6-well plates (1 × 10^3^ cells/well). Subsequently, gemcitabine, catechin, or their combination was added to the medium. After two weeks of incubation, the medium was removed and the colonies were fixed with 4% paraformaldehyde (Biosharp) for 20 min and then stained with 0.25% crystal violet (Servicebio, Wuhan, Hubei, China) for another 20 min. The colonies were photographed and counted.

### Animal model

The animal research was approved by the Experimental Animal Ethics Committee of the Medical School, Shenzhen University. Six-week-old female BALB/c nude mice were randomly divided into different groups (*n* = 5/group) and subcutaneously injected with 2 × 10^6^ indicated PC cells into the right axilla. Two weeks later, gemcitabine (100 mg/kg) alone, catechin (40 mg/kg) alone, or their combination was intraperitoneally injected twice a week. Saline served as a loading control. Calipers were used to detect the tumor size every 5 days, and the weights of the mice were recorded.

### Dual luciferase reporter assay

Cells were seeded on a plate and transiently transfected with the indicated plasmids when they reached 75% confluency. Luciferase activity was examined using the dual luciferase reporter assay kit (Promega, Madison, WI). Renilla luciferase activity was used as the loading control.

### Immunoprecipitation

The cells were washed and lysed, the proteins were collected, and primary antibodies were added to the lysis products and incubated overnight at 4 °C. Thereafter, sepharose-conjugated protein A + G beads (Beyotime) were added to the mixtures for another 4 h incubation. The beads were centrifuged, washed thoroughly, and boiled for 10 min. SDS-PAGE was conducted, and the proteins were analyzed by silver staining, western blotting, and mass spectrometry (MS).

### Immunofluorescence (IF) staining

MIA PaCa-2 and PANC-1 cells were seeded on coverslips in six-well plates. The cells were fixed in 4% paraformaldehyde for 20 min. Subsequently, a solution containing 5% normal goat serum and 0.25% Triton X-100 was used to block and permeabilize. Afterward, the coverslips were incubated with the indicated primary antibodies (Supplementary Table 1) overnight at 4 °C. The corresponding secondary antibodies were used, and PC cell nuclei were stained with DAPI the following day. Target protein expression was imaged.

### Chromatin immunoprecipitation (ChIP)

ChIP assay was performed using a kit (Millipore, Billerica, MA) according to the manufacturer’s instructions. The cell samples underwent successive formaldehyde cross-linking and glycine quenching. DNA fragments were obtained by sonication and subjected to IP. qPCR was performed to amplify the precipitated DNA products.

### GST pull down assay

GST agarose beads were used to bind with GST-tagged proteins. After washing with GST lysis buffer for two times, GST agarose beads were mixed with His-tagged proteins. Subsequently, the beads were washed five times with PBS and western blotting was performed.

### Ubiquitination assay

The cells were instantaneously transfected with different plasmids and MG132 was added to the medium. The cells were then harvested and lysed, and subsequent steps were performed in accordance with the IP assay. An in vitro ubiquitination assay was performed according to the manufacturer’s instructions. In short, the mixtures including His-tagged TRIM59/△RING, GST-tagged RBPJ, ubiquitin, reaction buffer, Mg^2+^-ATP solution, dH_2_O, and E1 and E2 enzymes were added to a 1.5 ml tube and mixed gently, then placed in a 37 °C water bath for 3 h incubation. Subsequently, western blotting was performed to evaluate the ubiquitination levels.

### Statistical analysis

Data were analyzed using SPSS software (version 23.0; Chicago, IL). Student’s t-test was used to evaluate differences between the groups, and multiple samples were subjected to one-way ANOVA. Log-rank test was performed on Kaplan–Meier analysis. Data are shown as mean ± standard deviation. Pearson’s coefficient was used to assess correlations. Statistical significance was set at *P* < 0.05.

## Results

### TRIM59 was identified as a key regulator of the Notch signaling pathway in PC

**Fig. 1:**
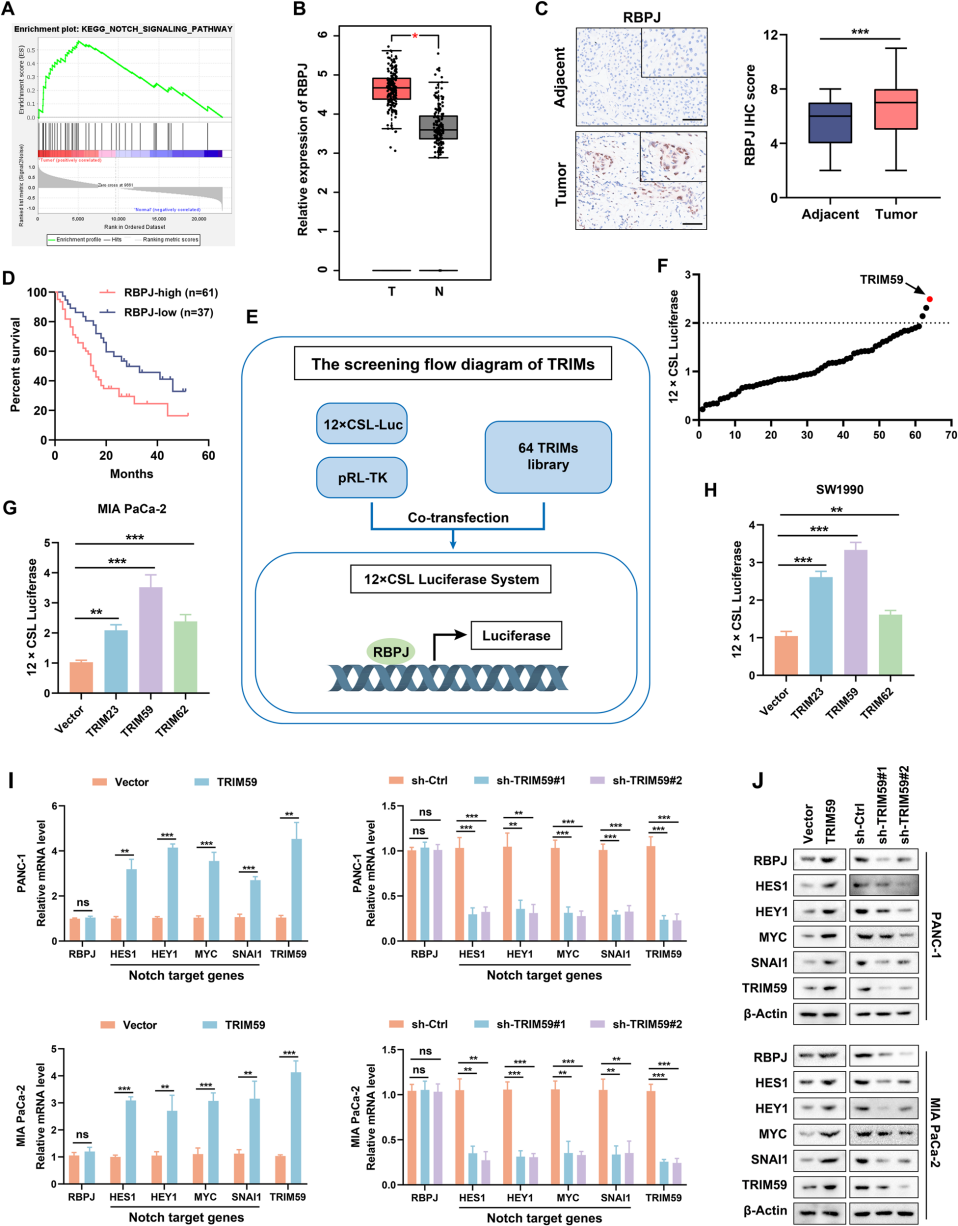
TRIM59 is identified as a key regulator of the Notch signaling pathway in PC. **A** The Notch signaling pathway was markedly altered in the PC dataset (GSE16515) of the GEO database verified by GSEA. **B** Relative expression level of RBPJ in the PC datasets of TCGA database. **C** RBPJ levels were assayed by IHC staining in PC and adjacent non-tumor tissues (*n* = 98). Scale bar, 100 μm. **D** The overall survival rate was assessed using Kaplan–Meier analysis with high/low RBPJ expression in PC patients. **E** Workflow of TRIM (s) screening using the 12× CSL-luciferase system. **F** Dual luciferase reporter assay was conducted to screen the TRIM candidates in PANC-1 cells. **G**, **H** The 12× CSL-luciferase activity was confirmed in MIA PaCa-2 (**G**) and SW1990 (**H**) cell lines using the dual luciferase reporter assay. **I**, **J** RT-qPCR (**I**) and western blotting (**J**) were performed to detect RBPJ, HES1, HEY1, MYC, SNAI1, and TRIM59 levels in PC cells. *P < 0.05, **P < 0.01, ***P < 0.001, ns no significance.

First, the PC dataset (GSE16515) from the Gene Expression Omnibus database was subjected to gene set enrichment analysis, which revealed that the Notch signaling pathway was dysregulated in PC (Fig. 1A). The relative expression level of the Notch signaling key TF RBPJ was upregulated in the PC datasets of The Cancer Genome Atlas (TCGA) database (Fig. 1B). The results of the IHC assay confirmed that the level of RBPJ was markedly elevated in PC tissues compared with that in adjacent non-tumor tissues (Fig. 1C). Furthermore, the Kaplan–Meier curve indicated that PC patients with higher RBPJ expression had a worse prognosis (Fig. 1D). To screen for the key TRIM (s) that regulate Notch signaling, a dual luciferase reporter assay was conducted, and we discovered that TRIM59 significantly enhanced Notch activity (Fig. 1E, F). Moreover, the TRIM59 group showed higher Notch activity than the control group in the other two PC cell lines (Fig. 1G, H). In addition, bioinformatic analysis revealed a positive correlation between TRIM59 expression and Notch signaling downstream target genes, including hes family bHLH TF 1 (HES1), hes-related family bHLH TF with YRPW motif 1 (HEY1), MYC proto-oncogene bHLH TF (MYC), and snail family transcriptional repressor 1 (SNAI1) (Fig. S1). Consistent with these results, TRIM59 overexpression increased and TRIM59 downregulation diminished the mRNA levels of HES1, HEY1, MYC, and SNAI1 in PC cells. However, the RBPJ mRNA levels did not change significantly when the expression of TRIM59 was altered (Fig. 1I). Intriguingly, TRIM59 upregulation and silencing increased and decreased, respectively, the protein levels of RBPJ, HES1, HEY1, MYC, and SNAI1 in PC cells (Fig. 1J). These data suggest that TRIM59 activates the Notch signaling pathway.

### TRIM59 was upregulated and led to a poor prognosis and gemcitabine efficacy in PC patients

**Fig. 2:**
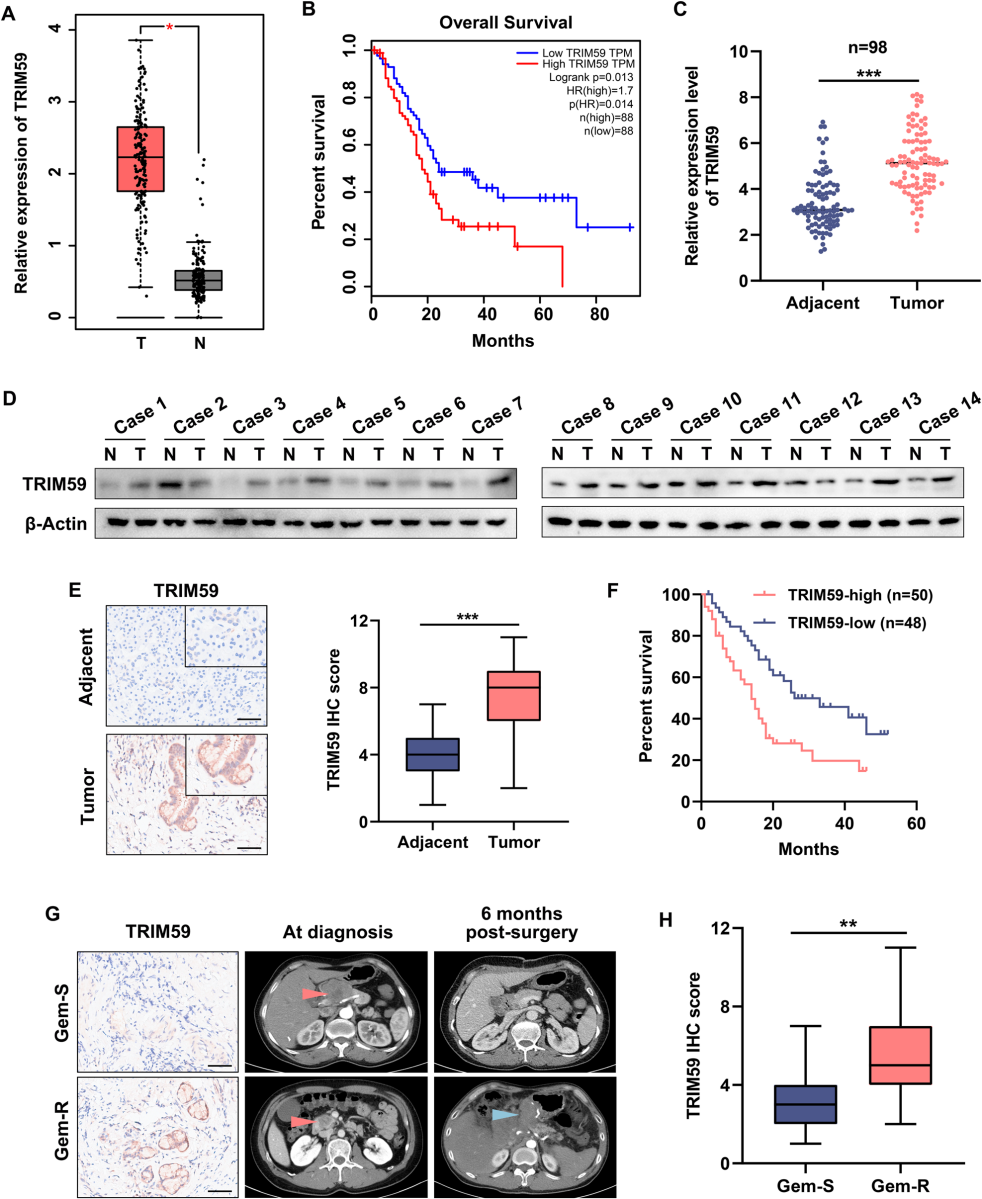
TRIM59 is upregulated and leads to a poor prognosis and gemcitabine efficacy in PC patients. **A** The mRNA levels of TRIM59 in the PC datasets obtained from TCGA database. **B** The overall survival plot from TCGA database of PC datasets with low vs. high TRIM59 levels. **C**–**E** RT-qPCR (**C**), western blot (**D**), and IHC (**E**) analyses of TRIM59 expression levels in PC and adjacent non-tumor tissues, respectively (*n* = 98). Scale bar, 100 μm. **F** Kaplan–Meier analysis of high/low TRIM59 expression in PC patients. **G** Representative IHC staining and computed tomography images of the gemcitabine-sensitive (Gem-S) and gemcitabine-resistant (Gem-R) PC patients. Scale bar, 100 μm. Red arrow, primary tumor; blue arrow, recurrent tumor. **H** IHC score of TRIM59 in the indicated PC specimens. *P < 0.05, **P < 0.01, ***P < 0.001.

Furthermore, by analyzing the data obtained from TCGA database, we discovered that the mRNA level of TRIM59 was considerably enhanced in PC samples compared with normal specimens and that PC patients with higher expression levels of TRIM59 had a worse survival outcome (Fig. 2A, B). Moreover, the results of RT-qPCR, western blotting, and IHC assays performed on PC specimens and adjacent non-tumor samples revealed an increase in TRIM59 expression in PC tissues (Fig. 2C–E). In addition, Kaplan–Meier analysis of clinical cases showed better survival in PC patients with low TRIM59 expression, whereas those with high TRIM59 expression exhibited the opposite result (Fig. 2F). Furthermore, the results of the IHC assay indicated that TRIM59 levels were remarkably elevated in the gemcitabine-resistant group than in the gemcitabine-sensitive group in PC patients who received postoperative gemcitabine treatment (Fig. 2G, H). In summary, these findings imply that TRIM59 may possess vital clinical value, especially the potential positive relationship between high TRIM59 expression levels and poor gemcitabine efficacy in PC.

### TRIM59 conferred gemcitabine resistance in PC in vitro and in vivo

**Fig. 3:**
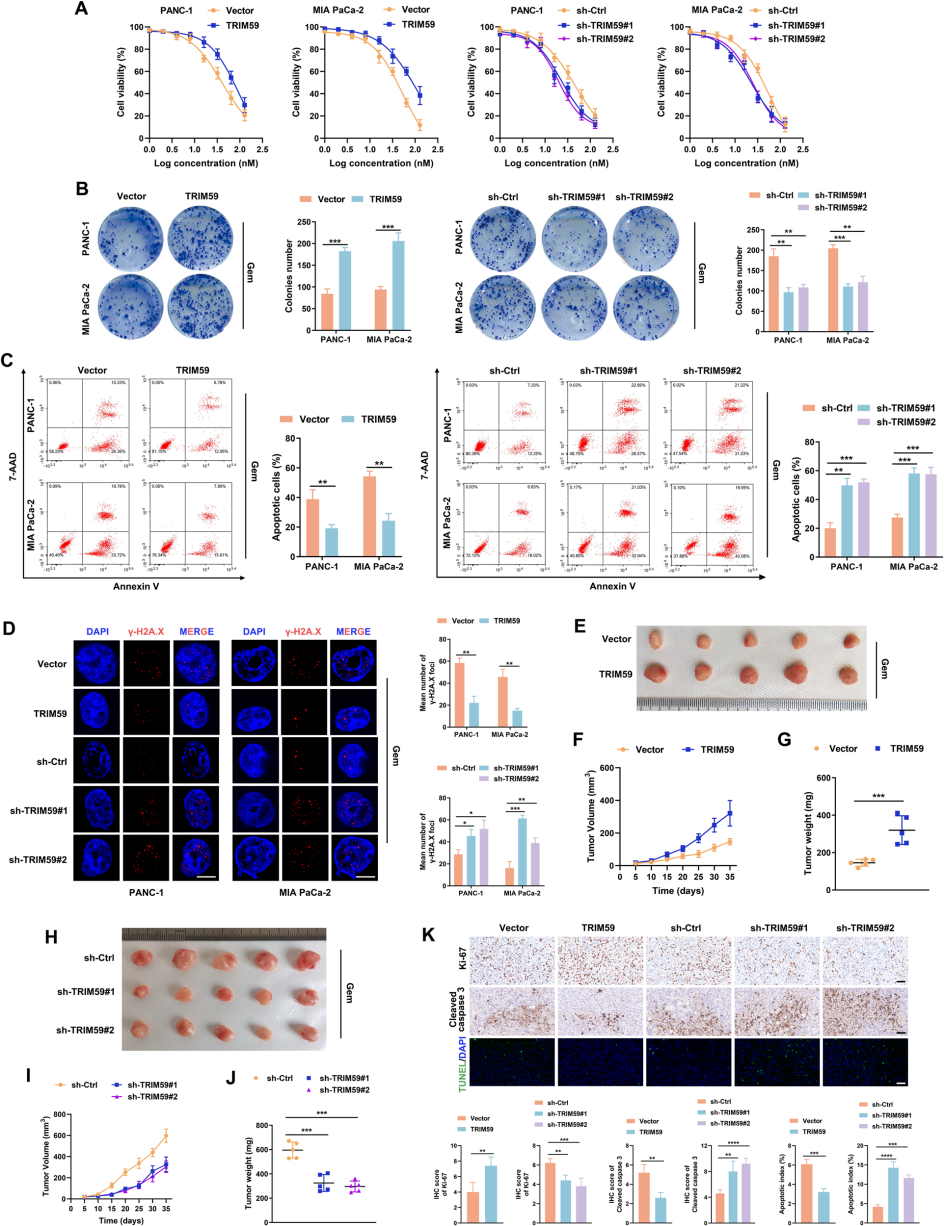
TRIM59 confers gemcitabine resistance in PC in vitro and in vivo. **A** Cell viability experiment was performed to determine the IC50 of gemcitabine in different groups of PC cells. **B**, **C** Colony formation (**B**) and flow cytometry (**C**) analyses of the indicated PC cells treated with gemcitabine. **D** Representative images of gemcitabine-induced γ-H2A.X foci in distinct PC cells. Scale bar, 5 μm. **E**–**J** Representative images of tumor-bearing mice in the indicated cells after gemcitabine treatment. Tumors from all mice (**E**, **H**) and their volume (**F**, **I**), and weight (**G**, **J**) are shown. **K** The tumor sections were subjected to IHC staining for Ki-67 and cleaved caspase 3 levels detection and TUNEL assay, respectively. Scale bar, 50 μm. *P < 0.05, **P < 0.01, ***P < 0.001, ****P < 0.0001.

To evaluate the role of TRIM59 in gemcitabine resistance in PC, PANC-1 and MIA PaCa-2 cells were treated with gemcitabine. The results of the cell viability assay showed that the IC50 of gemcitabine in PC cells increased or decreased when TRIM59 was upregulated or knocked down, respectively (Fig. 3A). Similarly, TRIM59 overexpression enhanced colony formation capacity, whereas TRIM59 silencing resulted in fewer colonies after gemcitabine treatment (Fig. 3B). Flow cytometry analysis demonstrated that the rate of apoptosis induced by gemcitabine was lower in TRIM59-overexpressing PC cells, whereas silencing of TRIM59 contributed to the opposite result (Fig. 3C). In addition, TRIM59 upregulation reduced the number of γ-H2A histone family member X (γ-H2A.X) foci induced by gemcitabine in PC cells; nevertheless, TRIM59 knockdown led to an increase in γ-H2A.X foci (Fig. 3D). To further determine whether the above in vitro results were in accordance with the function of TRIM59 in vivo, we performed an animal experiment and found that ectopic expression of TRIM59 substantially boosted tumor growth in mice treated with gemcitabine, whereas downregulation of TRIM59 sharply impeded tumor growth (Fig. 3E–J). Consistent with these findings, tumors formed by TRIM59-overexpressing PC cells showed high Ki-67 levels, low cleaved caspase 3 levels, and low apoptotic rates, as detected by IHC and TUNEL assays. However, TRIM59 silencing had the opposite effect (Fig. 3K). Overall, these findings suggested that TRIM59 conferred gemcitabine resistance in PC.

### TRIM59 interacted with and stabilized RBPJ in PC

**Fig. 4:**
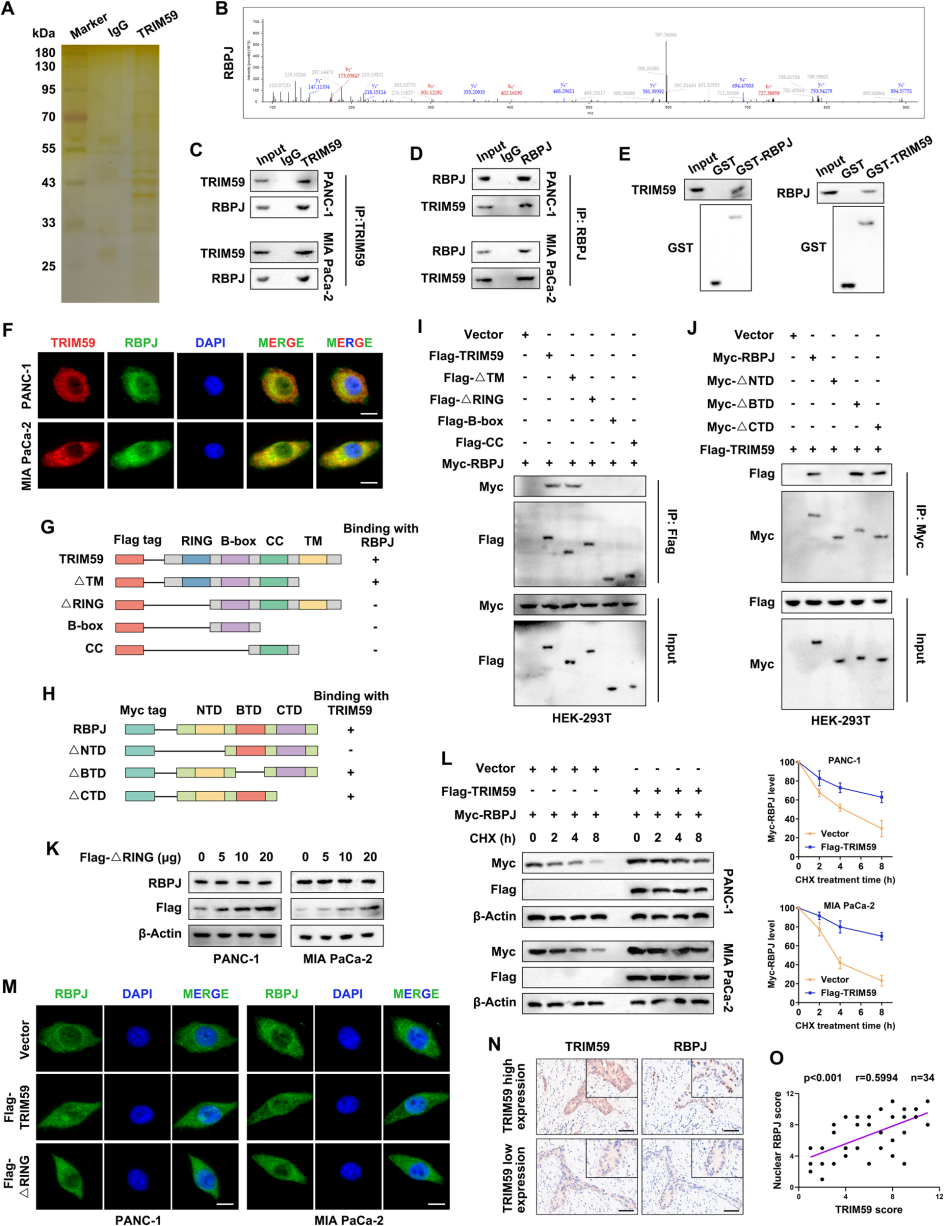
TRIM59 interacts with and stabilizes RBPJ in PC. **A** Silver staining assay. **B** MS analysis identified RBPJ as the interacting protein of TRIM59. **C**, **D** Co-IP experiment of TRIM59 (**C**) and RBPJ (**D**) in the indicated PC cells. **E** Interaction between TRIM59 and RBPJ was verified using GST pull-down assay. **F** IF staining showing the co-localization of TRIM59 and RBPJ. Scale bar, 10 μm. **G**–**J** Full-length and truncated plasmids of TRIM59 (**G**) and RBPJ (**H**) were transfected into HEK-293T cells. The binding site between TRIM59 (**I**) and RBPJ (**J**) was confirmed by Co-IP analysis. **K** Western blotting was performed to determine the protein levels of Flag-TRIM59 and RBPJ in PC cells transfected with the RING domain-deleted TRIM59 (Flag-△RING) plasmid. **L** CHX chasing assay in the indicated PC cells. **M** IF staining revealed the location of RBPJ. Scale bar, 10 μm. **N**, **O** IHC staining (**N**) and correlation analysis (**O**) of TRIM59 and RBPJ expression in PC tissues. Scale bar, 100 μm.

To investigate the mechanism underlying the increase in RBPJ protein levels mediated by TRIM59, a Co-IP assay was conducted. Silver staining and MS showed that RBPJ could bind to TRIM59 (Fig. 4A, B). Furthermore, IP analysis indicated an interaction between TRIM59 and RBPJ in PC cells (Fig. 4C, D). GST pull-down experiments also revealed that TRIM59 could bind to GST-RBPJ directly, and RBPJ could directly bind to GST-TRIM59 but not to GST (Fig. 4E). IF staining verified that TRIM59 and RBPJ were co-localized (Fig. 4F). Moreover, TRIM59 interacted with the NTD domain of RBPJ via the RING domain (Fig. 4G–J). Subsequently, the RING domain-deleted TRIM59 variant, which lacks E3 ligase activity, was transfected into PC cells, and RBPJ protein levels did not change significantly (Fig. 4K). Furthermore, cycloheximide chase assay showed that the protein half-life of RBPJ was prolonged after TRIM59 upregulation compared with that in the control group (Fig. 4L). IF staining showed that RBPJ levels in PC cell nuclei increased when TRIM59 was overexpressed, whereas the enzyme-inactive Mut of TRIM59 partly rescued this outcome (Fig. 4M). In addition, IHC staining and correlation analysis revealed a positive correlation between TRIM59 expression and nuclear RBPJ levels in PC tissues (Fig. 4N, O). Overall, these results indicated that TRIM59 interacts with RBPJ and increases its stability in PC.

### TRIM59 promoted K63-linked ubiquitination of RBPJ in PC

**Fig. 5:**
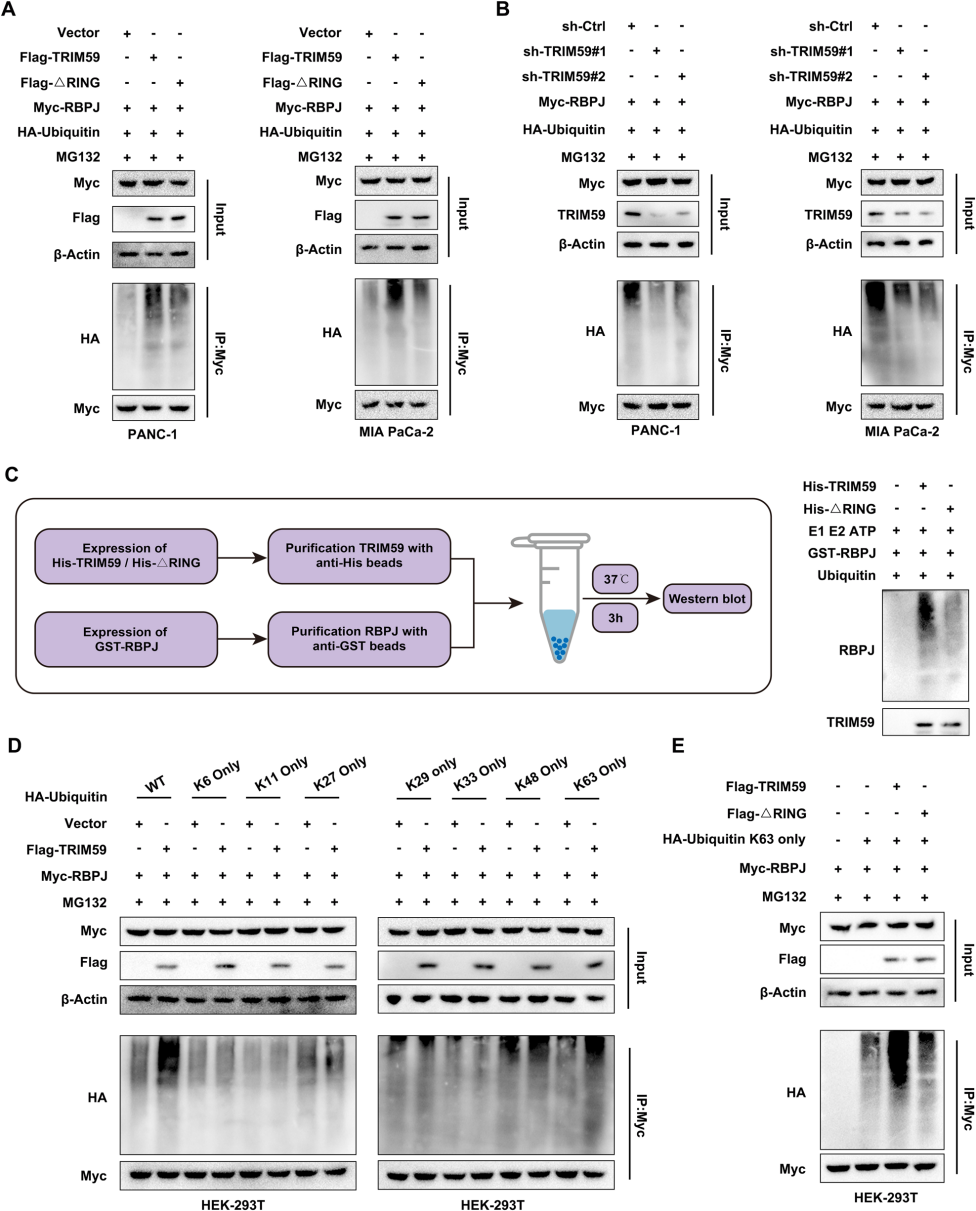
TRIM59 promotes the K63-linked ubiquitination of RBPJ in PC. **A**, **B** Ubiquitination assay was performed in the indicated cells. **C** In vitro ubiquitination assay. **D**, **E** The ubiquitination level of RBPJ in HEK-293T cells co-transfected with the indicated plasmids was examined.

Based on the E3 ubiquitin ligase characteristic of TRIM59 and inspired by the above findings, we attempted to determine whether the increased level of nuclear RBPJ induced by TRIM59 was mediated by ubiquitination. Ubiquitination assay revealed that the ubiquitination level of RBPJ was markedly elevated when TRIM59 was overexpressed, whereas the RING domain-deleted TRIM59 Mut partially reversed this effect (Fig. 5A). Conversely, TRIM59-silencing contributed to a reduction in the RBPJ ubiquitination levels (Fig. 5B). Purified TRIM59 augmented the ubiquitination level of RBPJ, whereas the enzyme-inactive TRIM59 Mut partially abrogated this result (Fig. 5C). Moreover, to explore which type of ubiquitin chain linkage was assembled by TRIM59 on RBPJ, Myc-tagged RBPJ along with the wild-type (WT) HA-tagged ubiquitin plasmid or the seven ubiquitin Muts, each of which contained only one lysine (K), were co-transfected into HEK-293T cells. The results of ubiquitination experiment indicated that TRIM59 boosted the assembly of K63-linked ubiquitin chains on RBPJ (Fig. 5D). Furthermore, consistent with these findings, TRIM59 overexpression enhanced the K63-linked ubiquitination of RBPJ and TRIM59 mutation partly recovered the increase in RBPJ K63-linked ubiquitination (Fig. 5E). Overall, these results suggest that TRIM59 facilitates the K63-linked ubiquitination of RBPJ in PC.

### RBPJ positively regulated TRIM59 expression at the transcriptional level

**Fig. 6:**
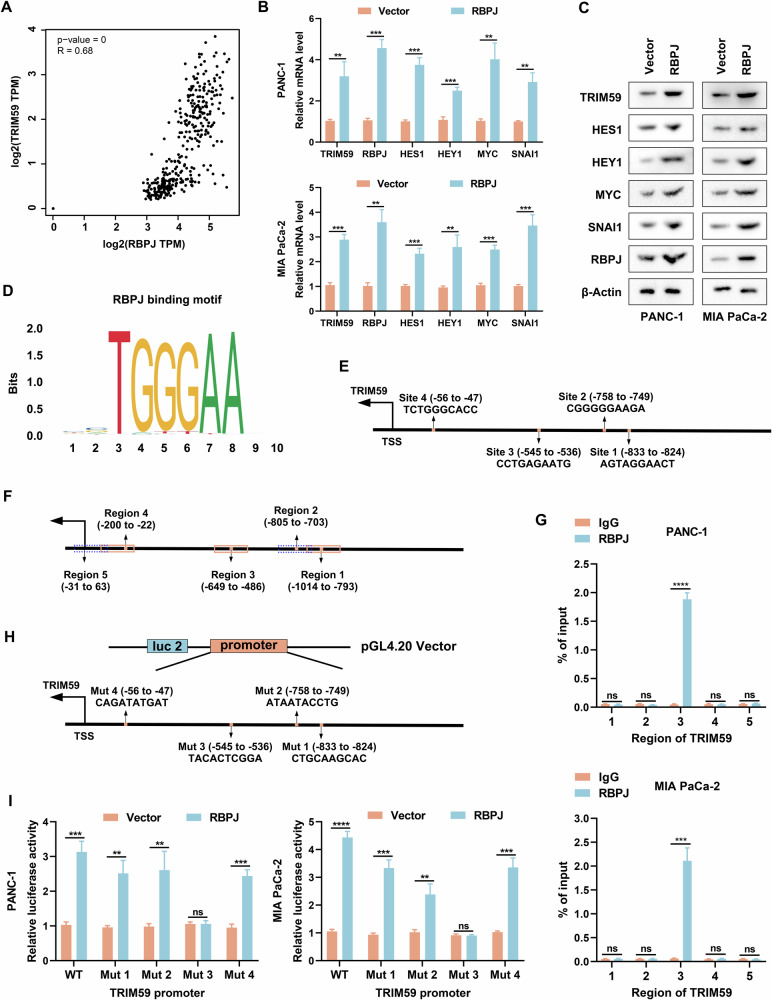
RBPJ positively modulates TRIM59 expression at the transcriptional level. **A** Correlation analysis between RBPJ and TRIM59 in the PC datasets from TCGA database. **B**, **C** RT-qPCR (**B**) and western blotting (**C**) were performed to probe the mRNA and protein levels of TRIM59, RBPJ, and Notch target genes. **D** The RBPJ-binding motif was acquired from the JASPAR database. **E** Schematic plot of the latent RBPJ-binding sites on the TRIM59 promoter. **F** Schematic illustration of primers designed for TRIM59 promoter regions. **G** Binding of RBPJ and the TRIM59 promoter was determined by ChIP-qPCR analysis. IgG was used as the negative control. **H** Schematic plot of the construction of dual luciferase reporter plasmid vector for the TRIM59 promoter. **I** Dual luciferase reporter assay was conducted on the indicated cells. **P < 0.01, ***P < 0.001, ****P < 0.0001, ns no significance.

The TF characteristics of RBPJ prompted us to examine whether TRIM59 was potentially regulated by RBPJ. Correlation analysis of the PC datasets in TCGA database showed that RBPJ was positively correlated with TRIM59 expression (Fig. 6A). Furthermore, both mRNA and protein levels of TRIM59 were notably elevated after RBPJ overexpression (Fig. 6B, C). We then acquired the RBPJ-binding motif from the JASPAR database (Fig. 6D), compared the promoter region sequence of TRIM59 with the RBPJ-binding motif, and found that there were four latent RBPJ-binding sites on the TRIM59 promoter (Fig. 6E). A ChIP assay was conducted and the corresponding qPCR primers were designed (Fig. 6F). ChIP-qPCR analysis revealed that the binding sequence of the TRIM59 promoter to RBPJ could be amplified by DNA precipitated by RBPJ (Fig. 6G). Moreover, the promoter sequences of TRIM59, which included WT and four underlying binding site Muts, were cloned into pGL4.20 plasmid, then transfected into control and RBPJ-upregulated PC cells, respectively (Fig. 6H). Thereafter, a dual luciferase reporter assay was performed and we found that RBPJ elevated TRIM59 promoter activity, whereas this effect was abolished when RBPJ binding site was mutated (Fig. 6I). Overall, our findings suggest that RBPJ regulates the transcription of TRIM59 by directly binding to its promoter.

### TRIM59 inhibitor catechin blocked the Notch signaling pathway and sensitized PC cells to gemcitabine

**Fig. 7:**
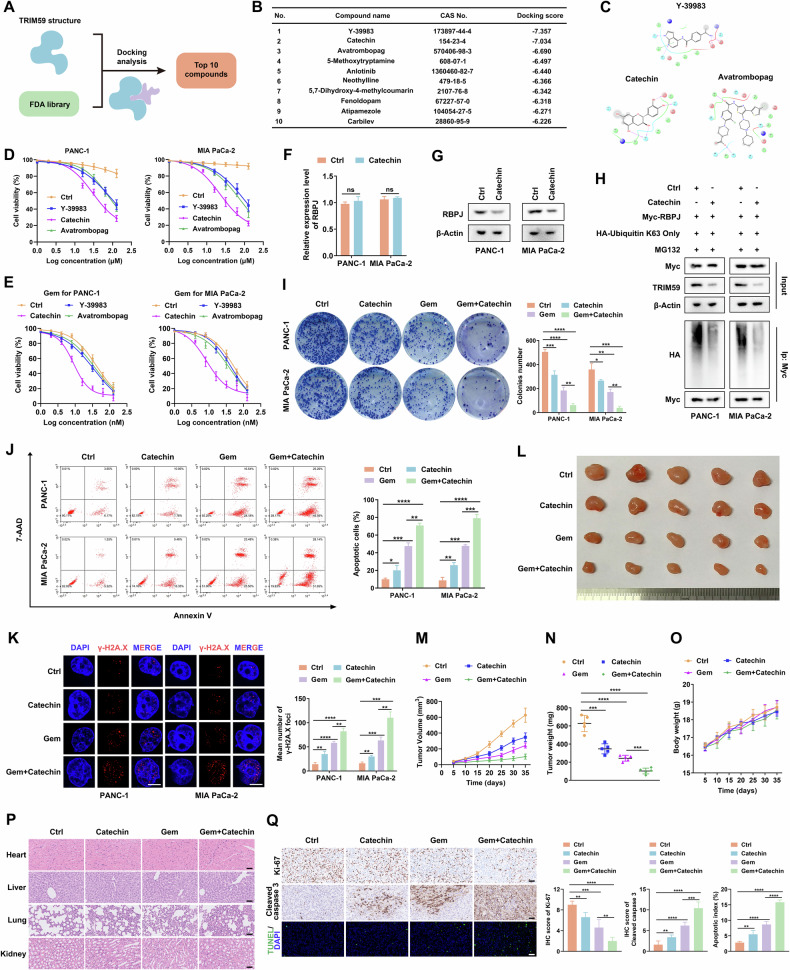
TRIM59 inhibitor catechin blocks the Notch signaling pathway and sensitizes PC cells to gemcitabine. **A** Virtual screening flowchart of the identification process of potential TRIM59 inhibitors. **B** Top 10 underlying TRIM59 inhibitors and their docking scores. **C** The acting force 2D images of Y-39983, catechin, and avatrombopag. **D** Cell viability assay of PC cells treated with Y-39983, catechin, and avatrombopag, respectively. **E** Cell viability assay was conducted in PC cells treated with gemcitabine plus Y-39983, catechin, and avatrombopag, respectively. **F**, **G** RT-qPCR (**F**) and western blotting (**G**) were performed to determine RBPJ levels in PC cells treated with/without catechin. **H** Ubiquitination assay was conducted in the indicated cells. **I**, **J** Colony formation (**I**) and flow cytometry (**J**) experiments were performed in distinctly treated groups of PC cells. **K** Representative images of γ-H2A.X foci in the indicated PC cells. Scale bar, 5 μm. **L**–**N** Representative images of mice tumors in different groups. Tumors from all mice (**L**), tumor volume (**M**), and tumor weight (**N**). **O** Alterations in the mice body weight over time. **P** The organs (heart, liver, lungs, and kidneys) obtained from the indicated mice were used for H&E staining. Scale bar, 50 μm. **Q** The tumor slices were subjected to IHC staining for Ki-67 and cleaved caspase 3 levels detection and TUNEL assay. Scale bar, 50 μm. *P < 0.05, **P < 0.01, ***P < 0.001, ****P < 0.0001, ns no significance.

The above studies strongly suggested that TRIM59 may serve as a vital biomarker for PC treatment. Next, to assess the implied application value of targeting TRIM59 as an effective method for treating PC, a structure-based virtual screening of nearly 3000 compounds was performed using the FDA library to identify potential TRIM59 inhibitors (Fig. 7A). The top 10 small-molecule compounds and their docking scores are shown in Fig. 7B. According to the docking score, the top 3 (Y-39983, catechin, and avatrombopag) compounds were selected, and the related acting force 2D images are shown in Fig. 7C. The results of the cell viability assay revealed that the IC50 of catechin was lower than that of Y-39983 and avatrombopag in PC cells (Fig. 7D), and only catechin enhanced the sensitivity of PC cells to gemcitabine (Fig. 7E). RT-qPCR showed that the mRNA levels of RBPJ were not significantly altered by catechin treatment (Fig. 7F). Nevertheless, RBPJ protein levels decreased significantly after culturing with catechin (Fig. 7G). In addition, western blotting and ubiquitination assays revealed that catechin inhibited the protein level of TRIM59 and reduced RBPJ K63-linked ubiquitination (Fig. 7H). The colony formation capacity was remarkably weakened when PC cells were treated with gemcitabine plus catechin, compared with gemcitabine alone (Fig. 7I). The combination of gemcitabine and catechin resulted in the highest apoptosis rate among the four groups (Fig. 7J). In addition, the number of γ-H2A.X foci in PC cells greatly increased in the combination group (Fig. 7K). In line with the in vitro results, tumor volume and weight were significantly decreased in mice treated with gemcitabine plus catechin (Fig. 7L–N). Notably, no significant weight loss was observed among the different groups of mice (Fig. 7O). H&E staining indicated that the major organs of the mice, including the heart, liver, lungs, and kidneys, did not show significant morphological alterations (Fig. 7P). Moreover, IHC showed that the combination of gemcitabine and catechin contributed to a substantial decrease in Ki-67 expression and enhanced cleaved caspase 3 levels. Additionally, TUNEL staining indicated that the number of apoptotic PC cells was drastically elevated in the group that received the combination therapy (Fig. 7Q). Overall, these results imply that catechin could significantly increase the sensitivity of PC cells to gemcitabine.

## Discussion

Chemoresistance may be a major cause of death in PC patients; however, the potential molecular mechanism underlying this clinical outcome remains unclear. Thus, clarifying the molecules and modulatory processes underlying PC chemoresistance may contribute to the development of novel strategies to treat this intractable cancer. According to a recent study, Notch signaling is involved in PC chemoresistance [22]. Previously, we demonstrated that the Notch signaling pathway is activated, which contributes to enhanced proliferation and mobility in PC [23]. In addition, we also discovered that the vital members of the TRIM family proteins, TRIM31 and TRIM37, confer gemcitabine and fluorouracil resistance in PC by activating the NF-κB and AKT–GSK-3β–β-catenin signaling pathways, respectively [13, 14]. However, little is known about PC chemoresistance with respect to Notch signaling and TRIM proteins. In this study, we identified TRIM59 as an innovative E3 ubiquitin ligase that activates Notch signaling in PC cells. A previous study revealed that TRIM59 is highly expressed and contributes to gefitinib resistance in lung cancer [12]. Similarly, we confirmed that TRIM59 levels were increased in PC samples and were positively related to poor prognosis and gemcitabine resistance in PC patients. We further verified that TRIM59 facilitated gemcitabine resistance in PC cells. Moreover, to explore the mechanism underlying the increase in RBPJ protein levels mediated by TRIM59, an interaction analysis between TRIM59 and RBPJ was performed. We found that TRIM59 interacted with and stabilized RBPJ, which relied on the E3 ubiquitin ligase activity of TRIM59. Based on these results, we determined whether the enhanced stabilization of RBPJ by TRIM59 was mediated by ubiquitination.

Ubiquitination is a pivotal post-translational modification that plays a critical role in modulating various cellular processes by governing protein interactions, localization, trafficking, and stability [24]. Seven lysine (K) residues (K6, K11, K27, K29, K33, K48, and K63) are present in ubiquitin. Various ubiquitin chain linkages are assembled by these lysine residues, and the ubiquitin chain linkages have different biological effects on life entities [25–27]. K48-linked ubiquitination plays a role in the induction of protein degradation [28], while the K63-linked ubiquitin chain acts as a non-degradative signal to stimulate the immune response and NF-κB pathway [29]. Recently, Xiong et al. demonstrated that the E3 ubiquitin ligase TRAF6 enhances the protein level of PD-L1 by facilitating K63-linked ubiquitination [30]. Another team discovered that TRIM31 interacts with p53 and mediates K63-linked ubiquitination of p53 via its RING domain, leading to p53 stabilization and activation [31]. Kolapalli et al. reported that the E3 ubiquitin ligase DZIP3 enhanced K63-linked ubiquitination and stability of cyclin D1 via its RING domain, thus leading to tumor progression [32]. However, the effect of TRIM59 ubiquitination on RBPJ has not yet been clarified. To this end, ubiquitination assays were performed, and the results showed that TRIM59 specifically enhanced the K63-linked ubiquitination of RBPJ, whereas the RING domain-deleted TRIM59 variant, which lacks E3 ubiquitin ligase activity, partly rescued this effect.

TFs are a class of proteins that regulate cellular gene expression. They play a critical role in coordinating transcription, thus affecting cell fate [33]. Given that RBPJ is a key TF in the regulation of the Notch signaling pathway [34], we conducted further experiments and found that RBPJ transcriptionally upregulated TRIM59 expression. We initially demonstrated that TRIM59 increased RBPJ levels; thus, a positive feedback TRIM59/RBPJ loop was formed in PC.

Based on the aforementioned results, TRIM59 holds great potential for PC treatment and may serve as a promising target for overcoming gemcitabine resistance. Owing to the lack of known TRIM59 inhibitors, we identified a small-molecule compound, catechin, via virtual screening, which has the potential to act as a TRIM59 inhibitor. Catechin is a major phenolic constituent of green tea [35] and is reported to overcome drug resistance in PC cells [36]. In our study, we proved that catechin sensitized PC cells to gemcitabine, and most significantly, that the combination of gemcitabine and catechin was safe and well-tolerated in vivo at effective doses.

**Fig. 8:**
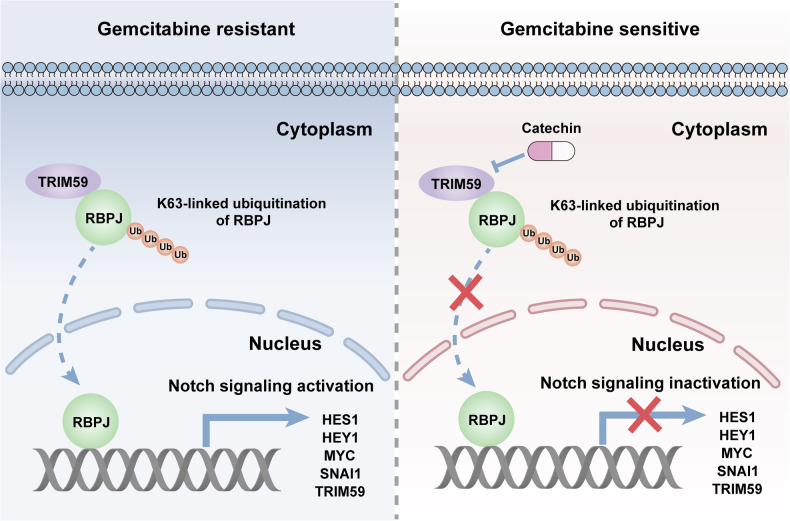
Schematic diagram of our study.

In summary, our study showed that a novel E3 ubiquitin ligase, TRIM59, which activates the Notch signaling pathway, was upregulated in PC. TRIM59 interacted with RBPJ and promoted K63-linked ubiquitination, leading to its increased stabilization. RBPJ positively modulated TRIM59 expression at the transcriptional level through its TF characteristics, and TRIM59 and RBPJ formed a positive feedback loop to confer gemcitabine resistance in PC. Finally, we discovered that the TRIM59 inhibitor catechin sensitized PC cells to gemcitabine treatment (Fig. 8). This study provides a potential strategy for improving the efficacy of gemcitabine in gemcitabine-resistant PC patients.

## Supplementary information

Supplementary Figure 1

Supplementary Figure 2

Supplementary Figure 3

Supplementary Figure 4

Supplementary Figure 5

Supplementary Table 1

Supplementary Table 2

Supplementary Table 3

Original Western Blots

## Data Availability

The data of this study are available from the corresponding author upon reasonable request.
