# Supplementary material for: TRIM59/RBPJ positive feedback circuit confers gemcitabine resistance in pancreatic cancer by activating the Notch signaling pathway
Source: Cell Death Dis. 2024 Dec 26;15(12):932. doi: 10.1038/s41419-024-07324-y (PMC11671593; doi:10.1038/s41419-024-07324-y)
Supplement: Supplementary file 1 — Supplementary Figure 1 [file 41419_2024_7324_MOESM1_ESM.docx]

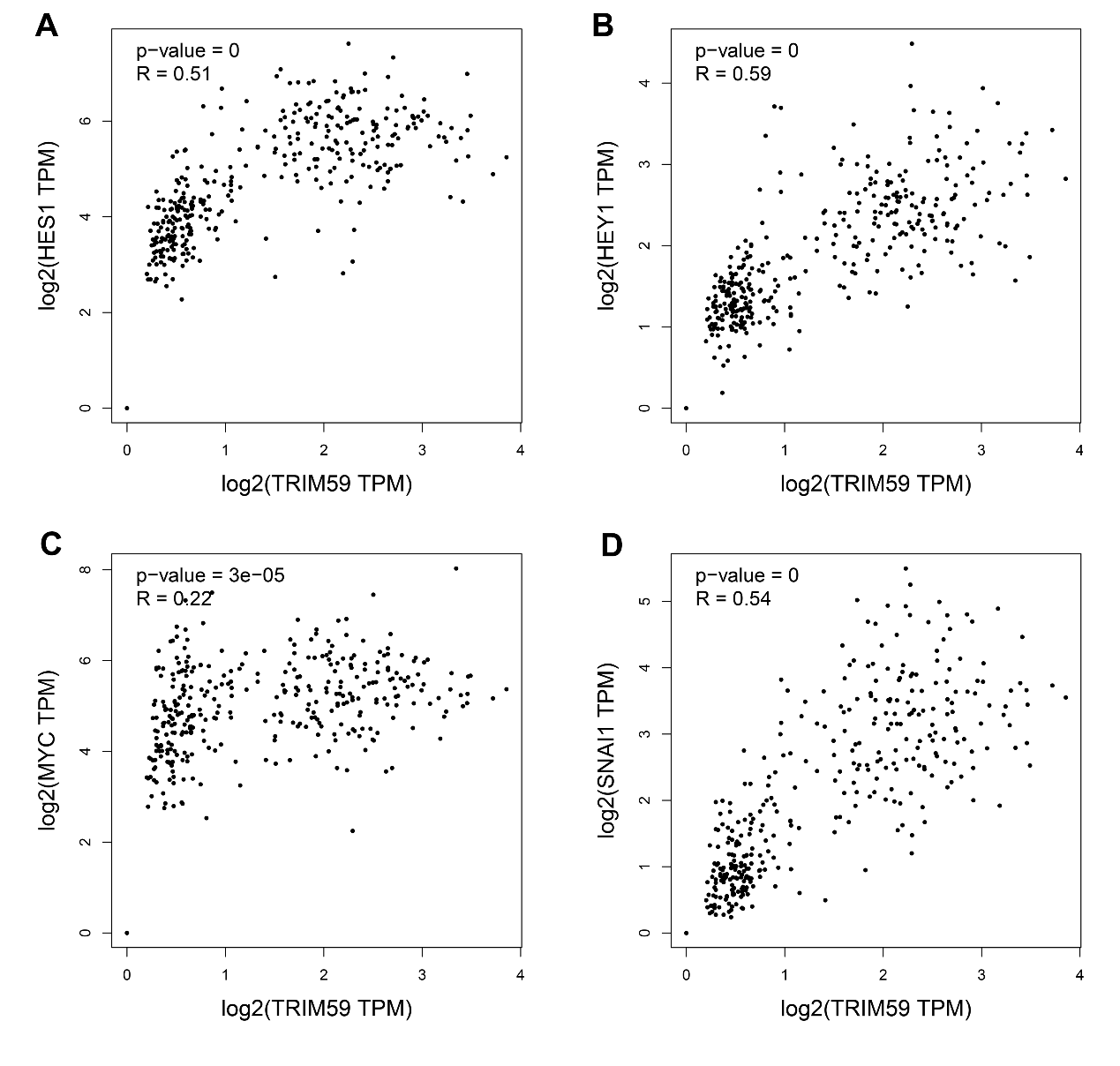


**Figure S1. Expression correlation analysis.** (**A-D**) The expression correlation between TRIM59 and HES1 (**A**), HEY1 (**B**), MYC (**C**), and SNAI1 (**D**) was analyzed in the PC datasets of TCGA database via the Gene Expression Profiling Interactive Analysis (GEPIA) website.
