# Supplementary material for: TRIM59/RBPJ positive feedback circuit confers gemcitabine resistance in pancreatic cancer by activating the Notch signaling pathway
Source: Cell Death Dis. 2024 Dec 26;15(12):932. doi: 10.1038/s41419-024-07324-y (PMC11671593; doi:10.1038/s41419-024-07324-y)
Supplement: Supplementary file 2 — Supplementary Figure 2 [file 41419_2024_7324_MOESM2_ESM.docx]

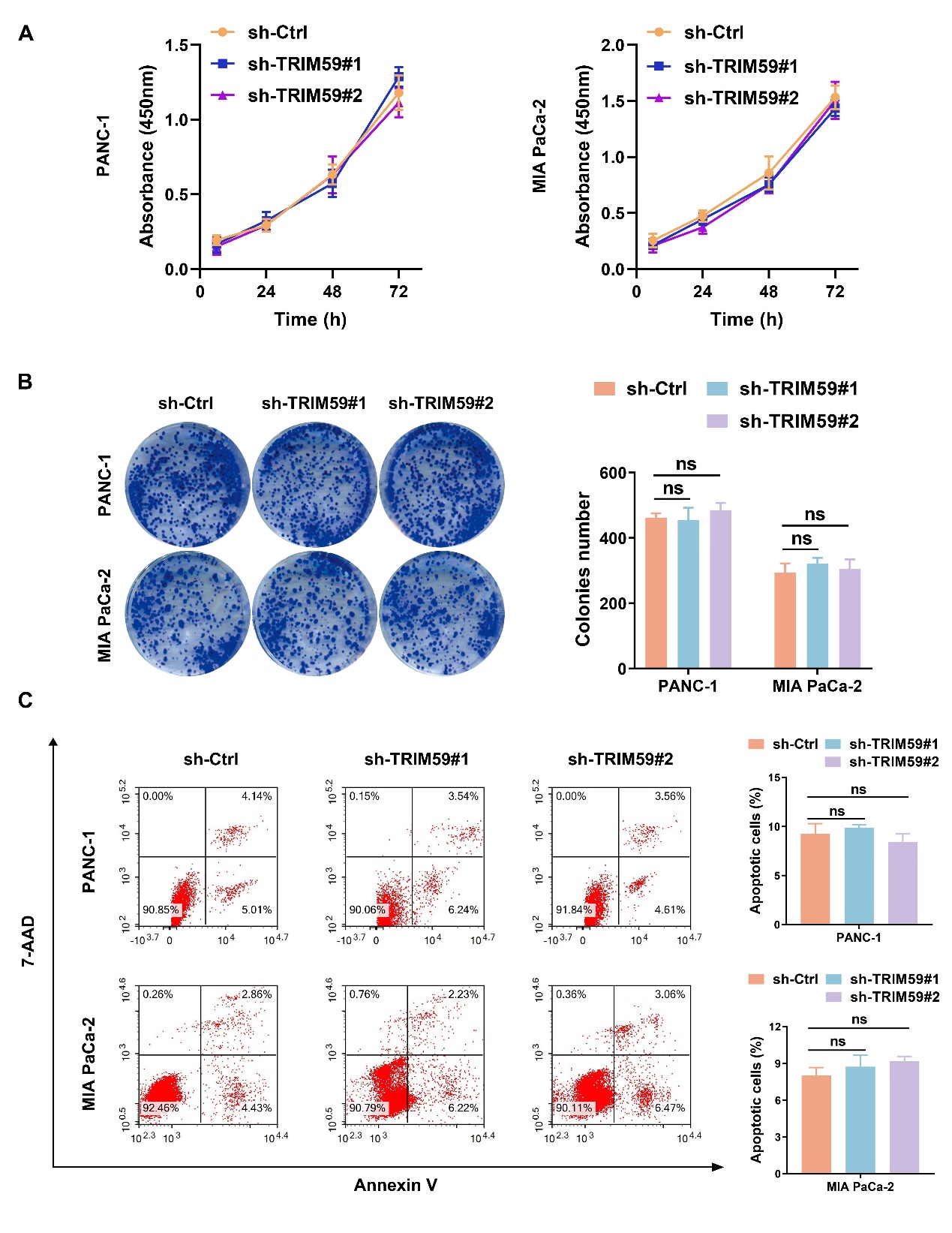


**Figure S2. TRIM59 doesn’t play a role in the proliferation and apoptosis of PC cells.** (**A-C**) CCK-8 (**A**), colony formation (**B**), and flow cytometry (**C**) experiments were used to evaluate the proliferation and apoptosis of the indicated PC cells, respectively. ns: no significance
