# Supplementary figures and images for: TRIM59/RBPJ positive feedback circuit confers gemcitabine resistance in pancreatic cancer by activating the Notch signaling pathway
Source: Cell Death Dis. 2024 Dec 26;15(12):932. doi: 10.1038/s41419-024-07324-y (PMC11671593; doi:10.1038/s41419-024-07324-y)

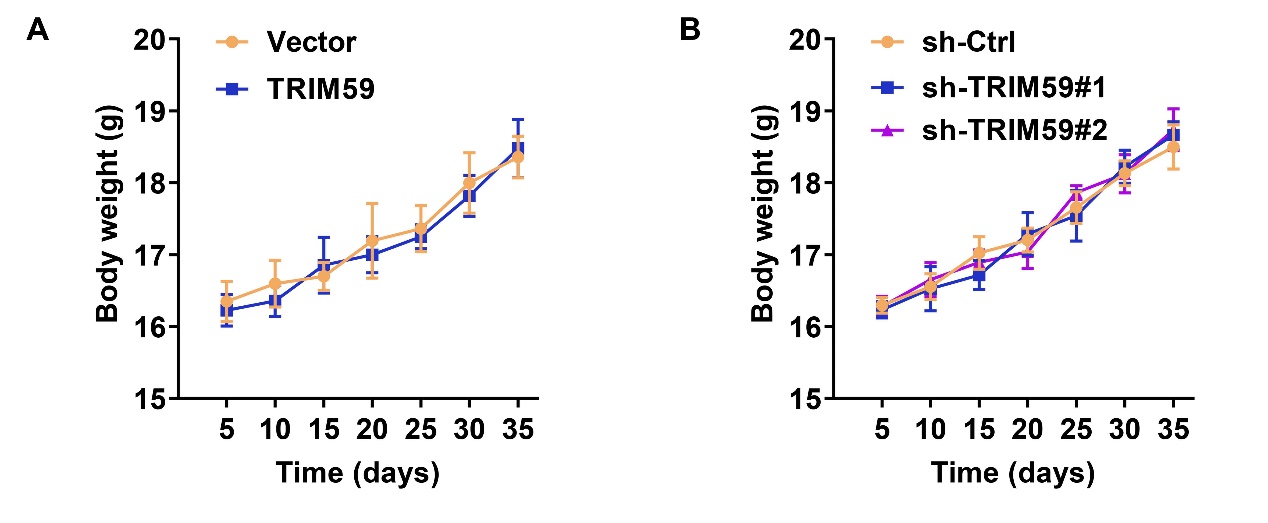


**Figure S3.** The alterations in the mice body weight over time in the indicated groups (**A, B**).

Supplement: Supplementary file 3 — Supplementary Figure 3 [file 41419_2024_7324_MOESM3_ESM.docx]

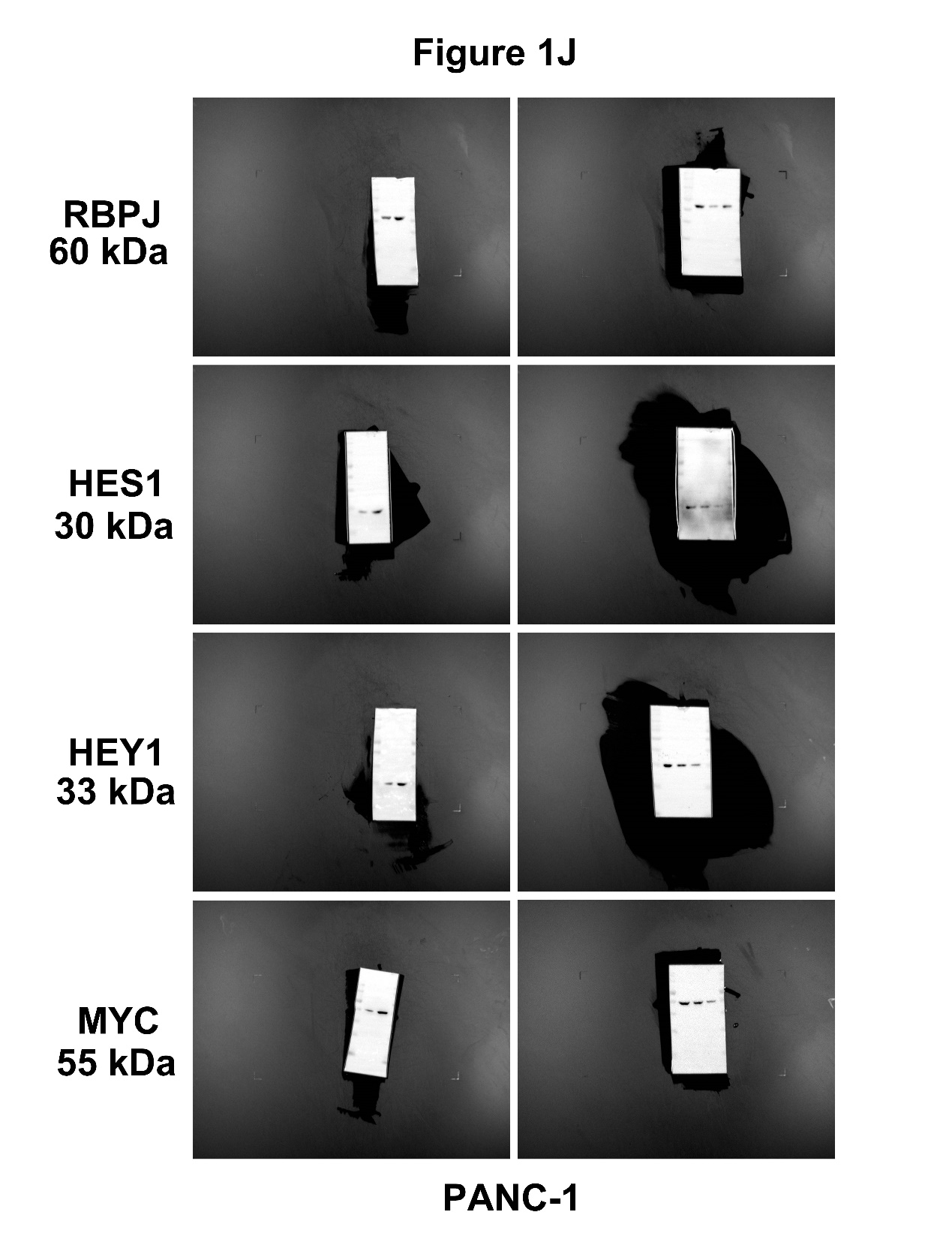


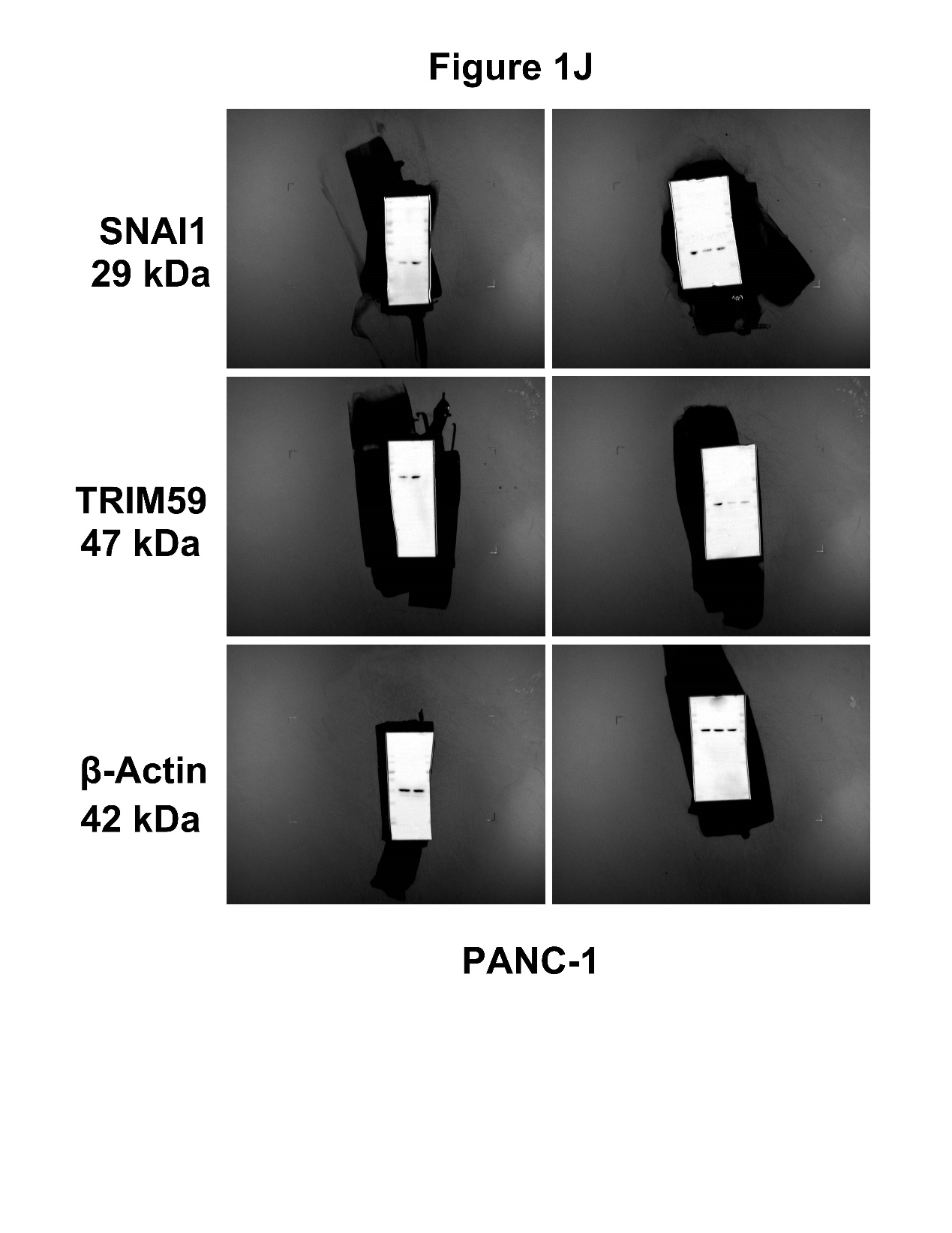


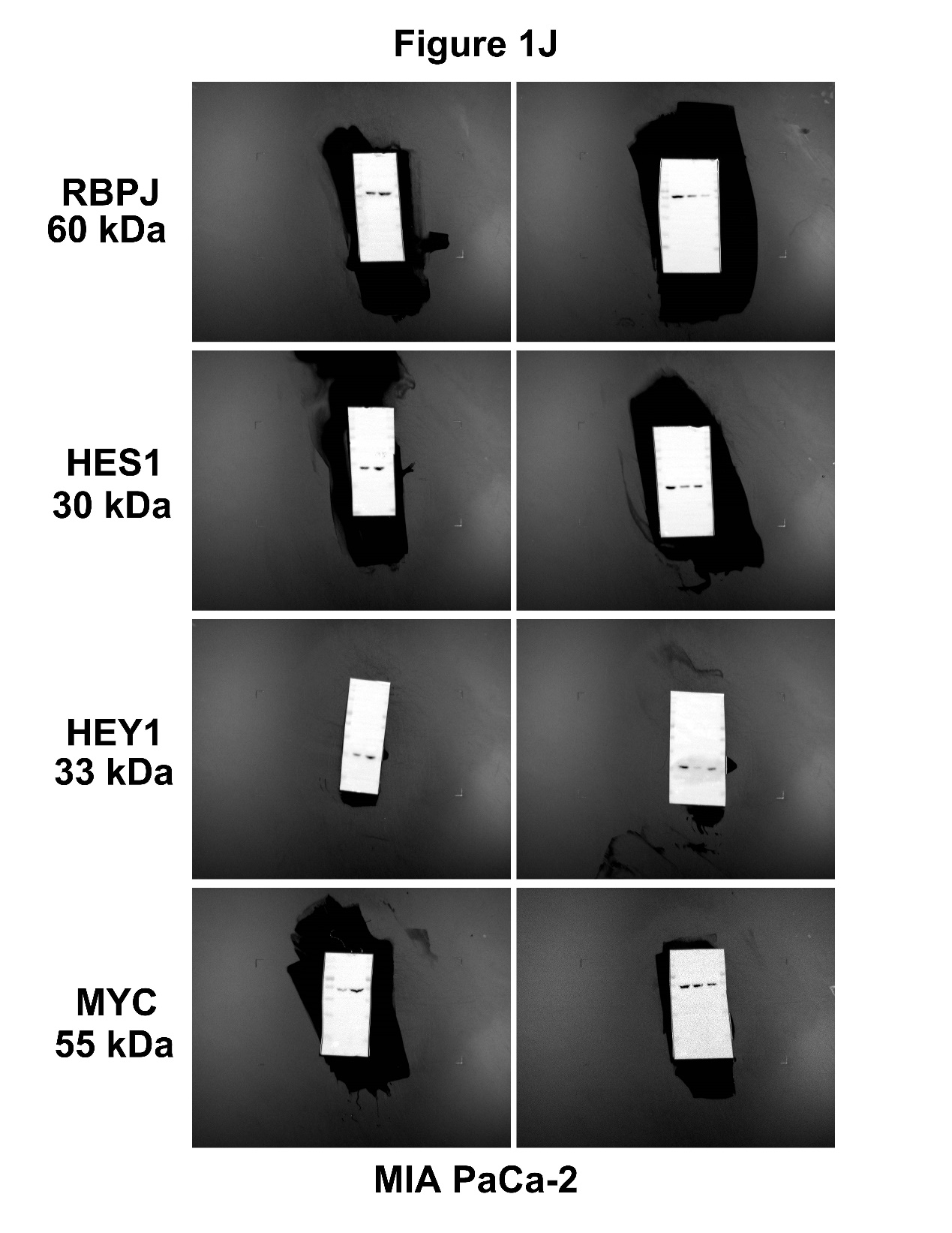


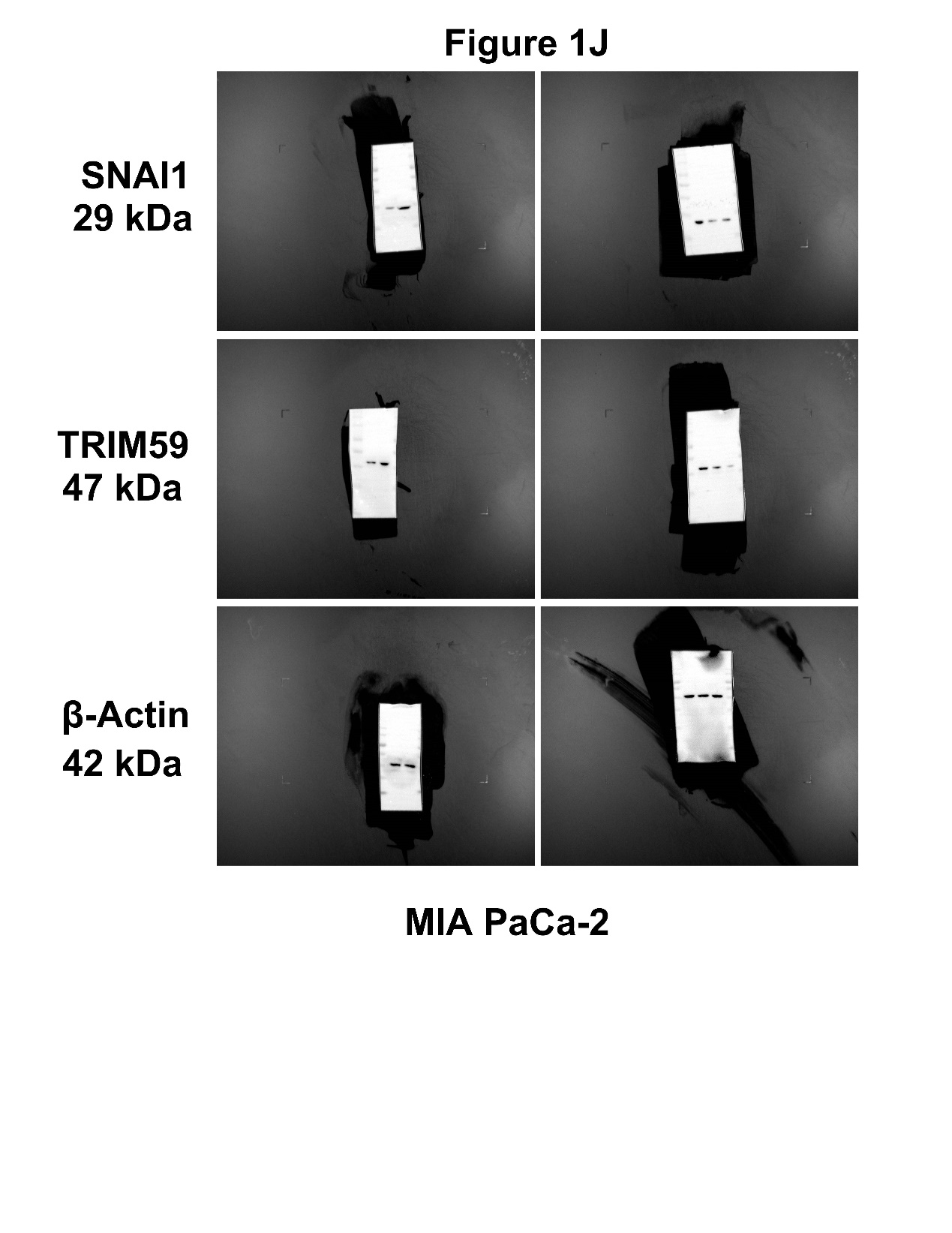


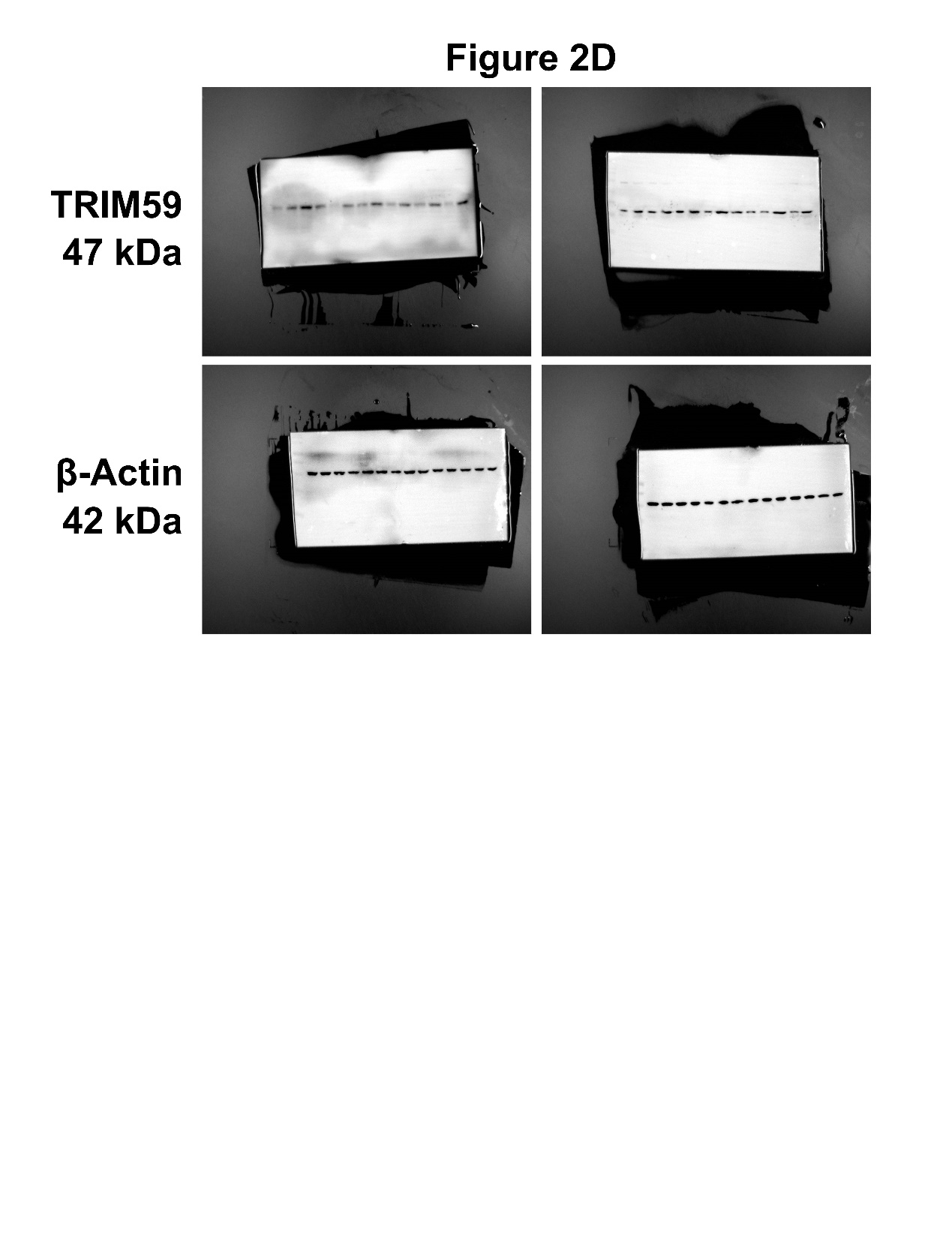


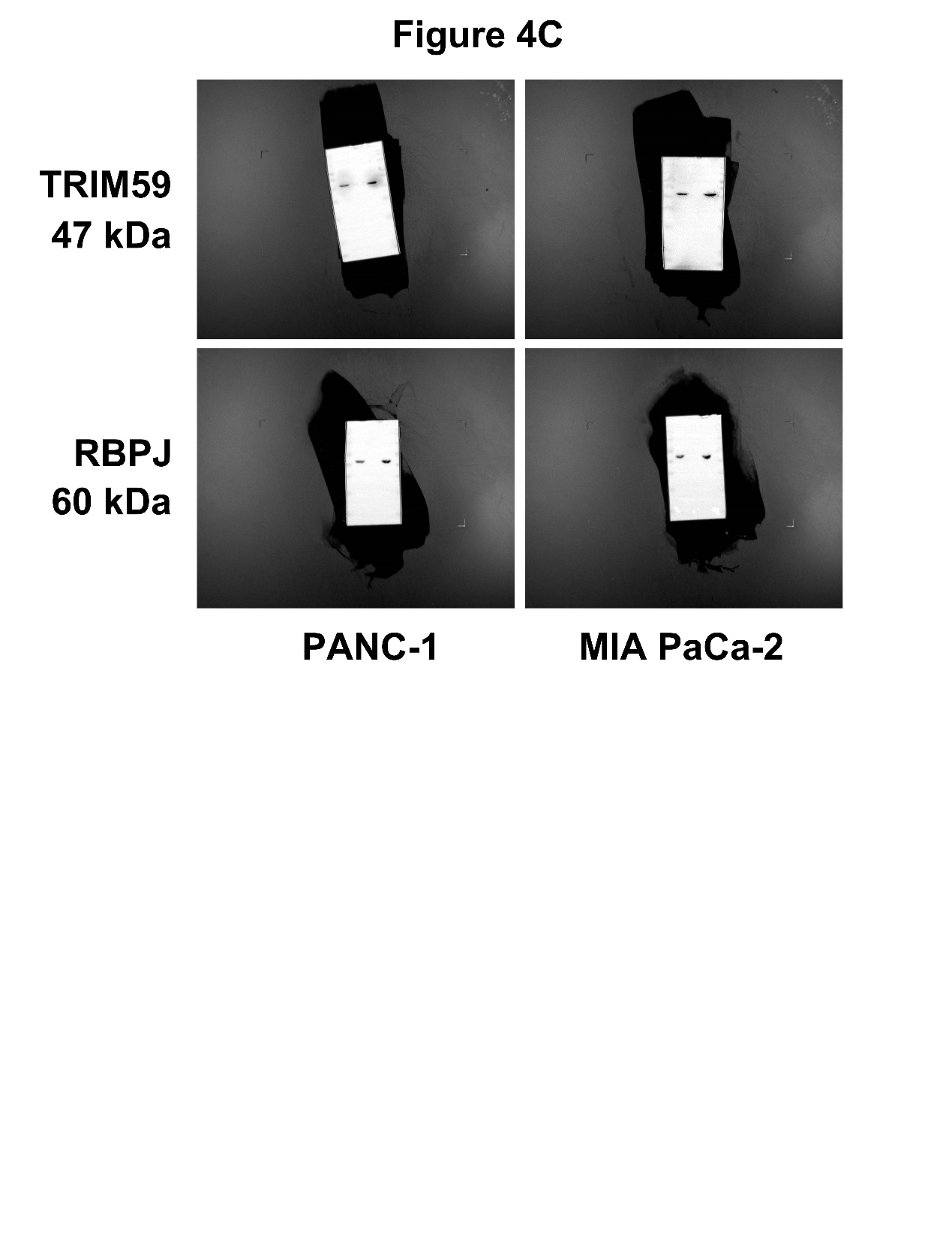


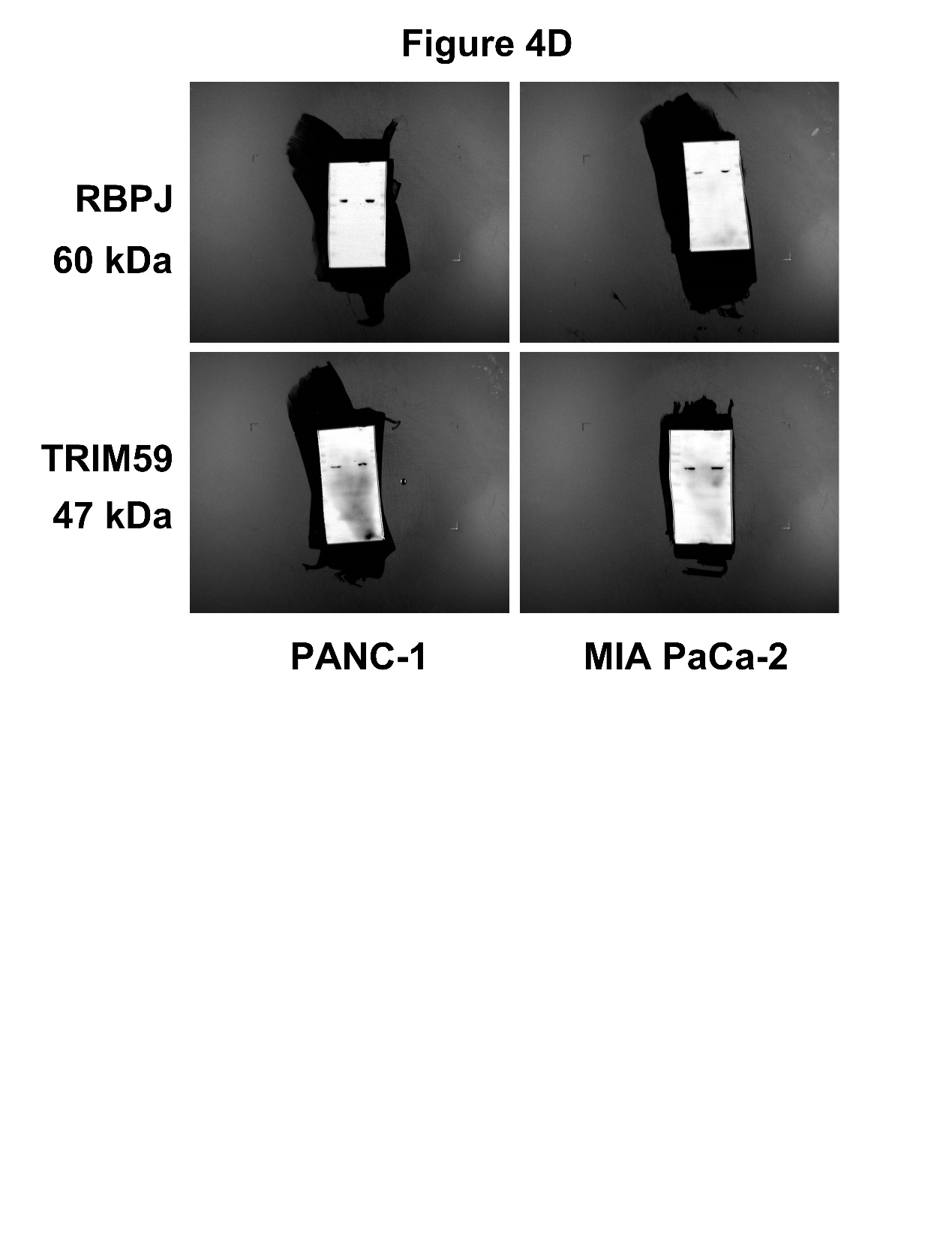


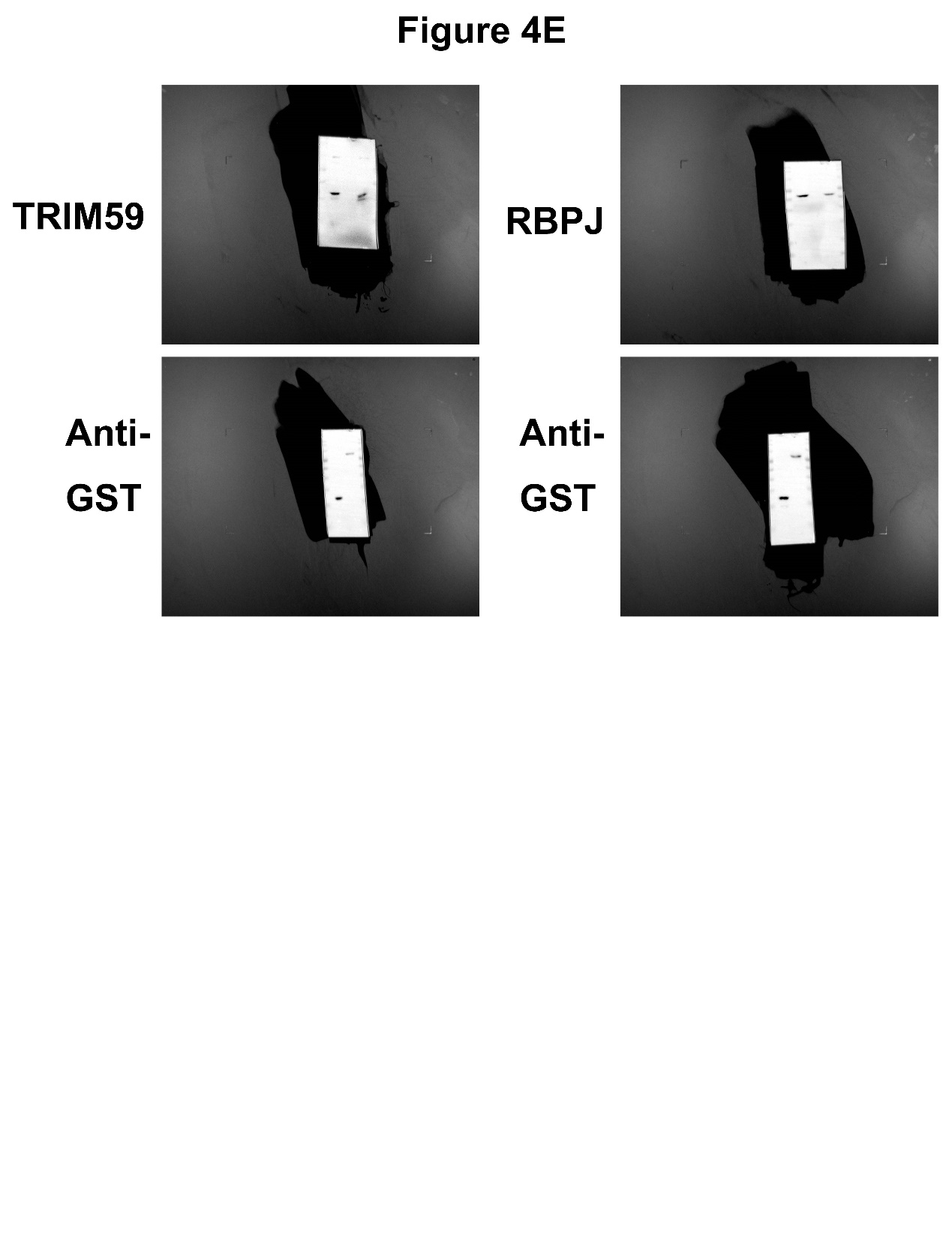


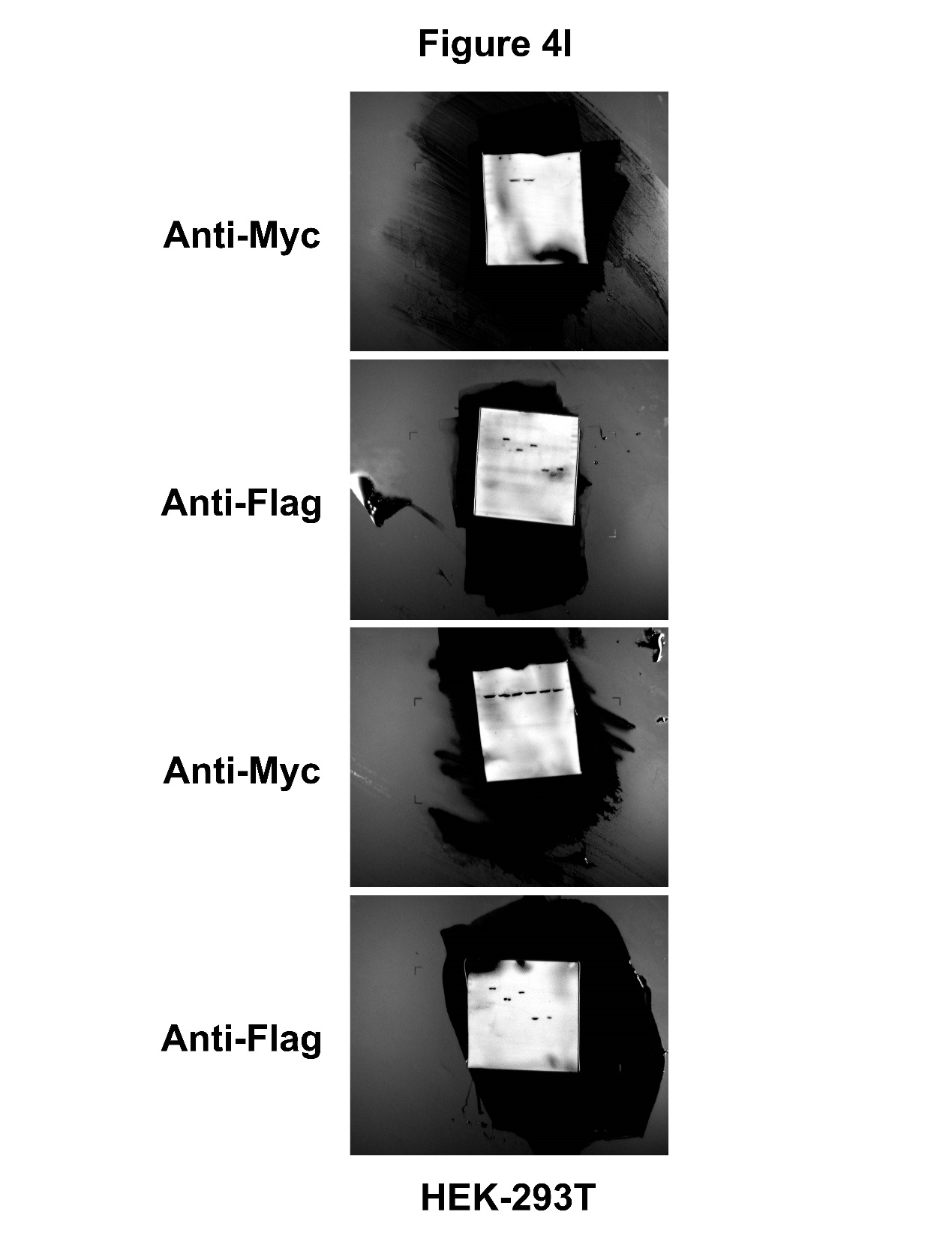


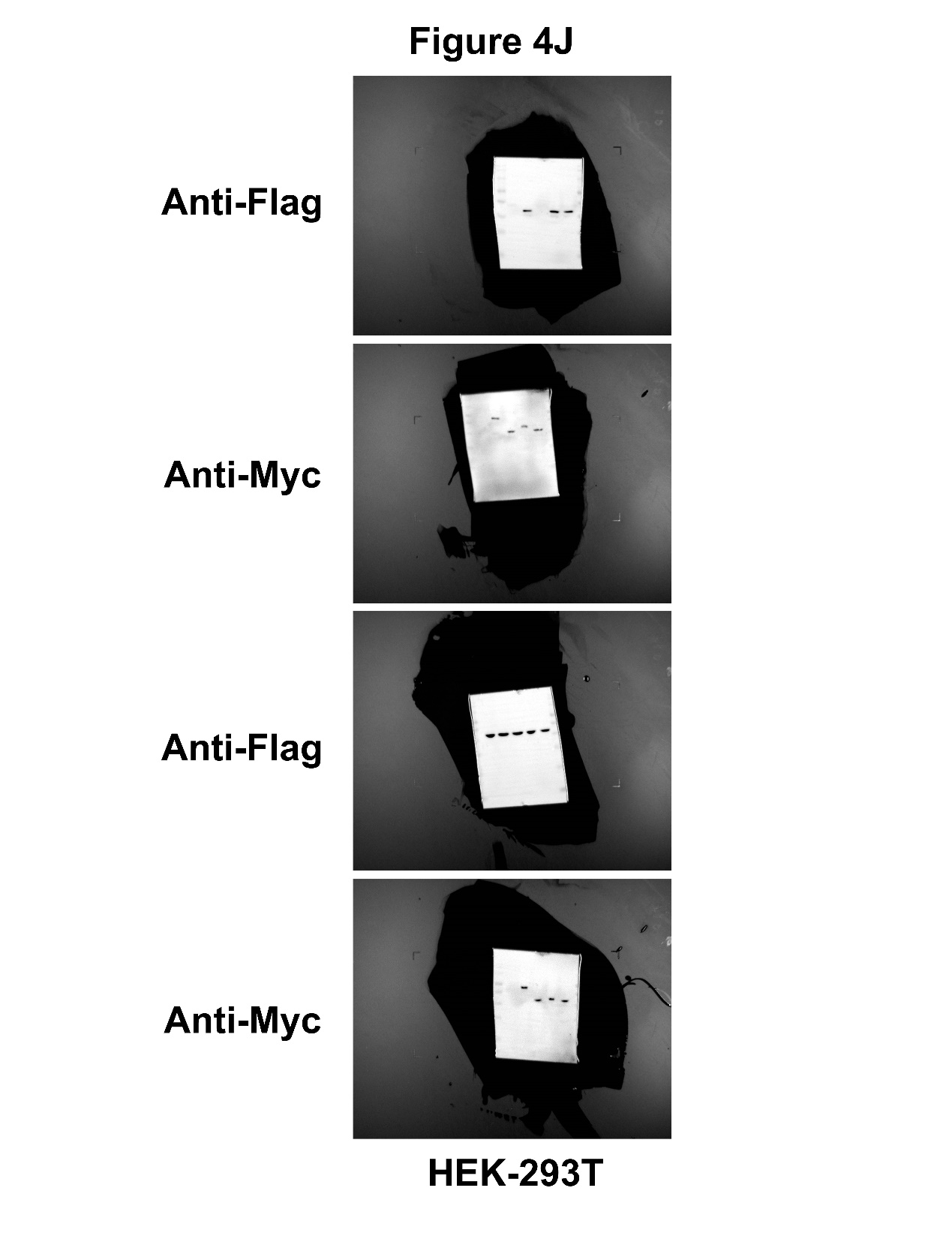


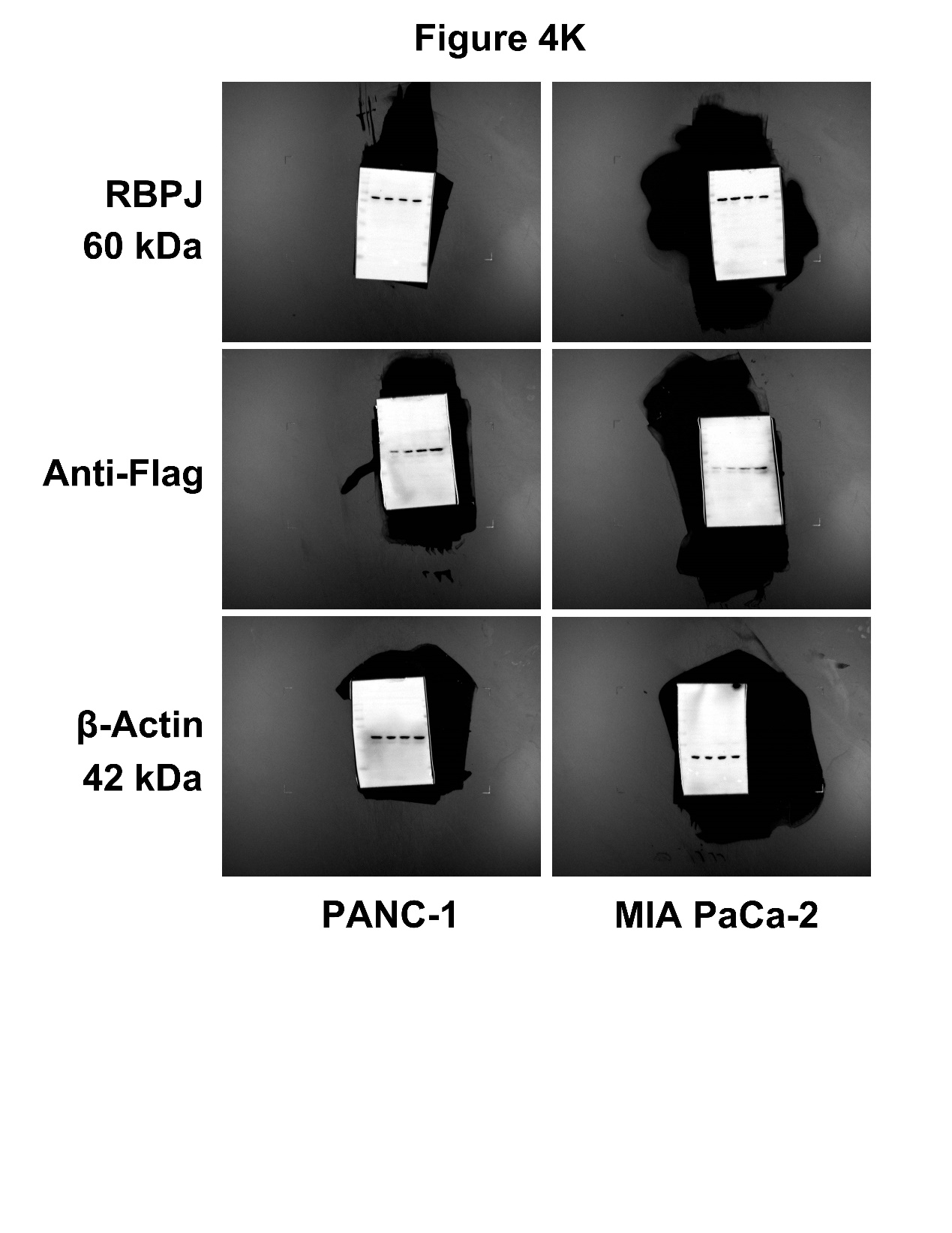


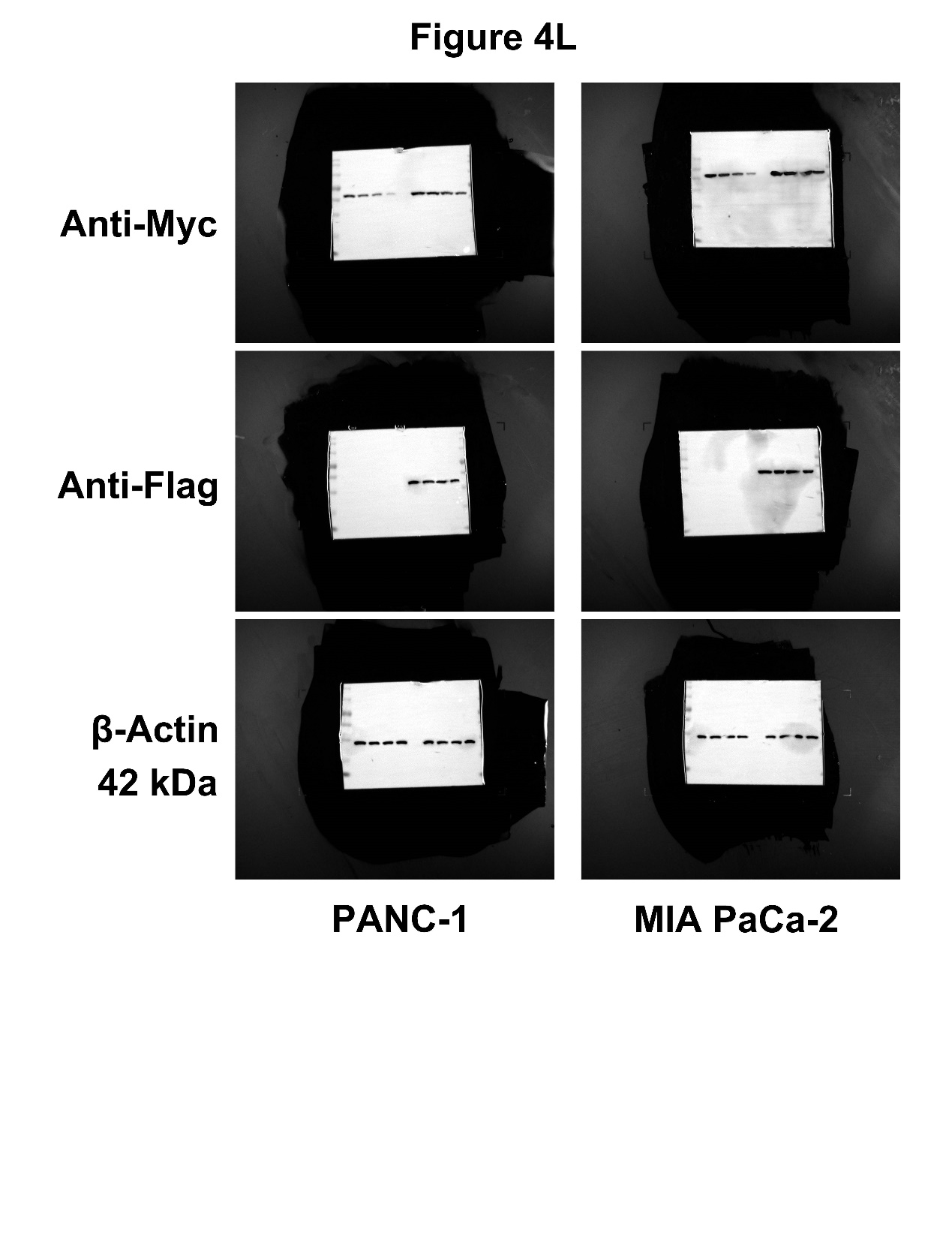


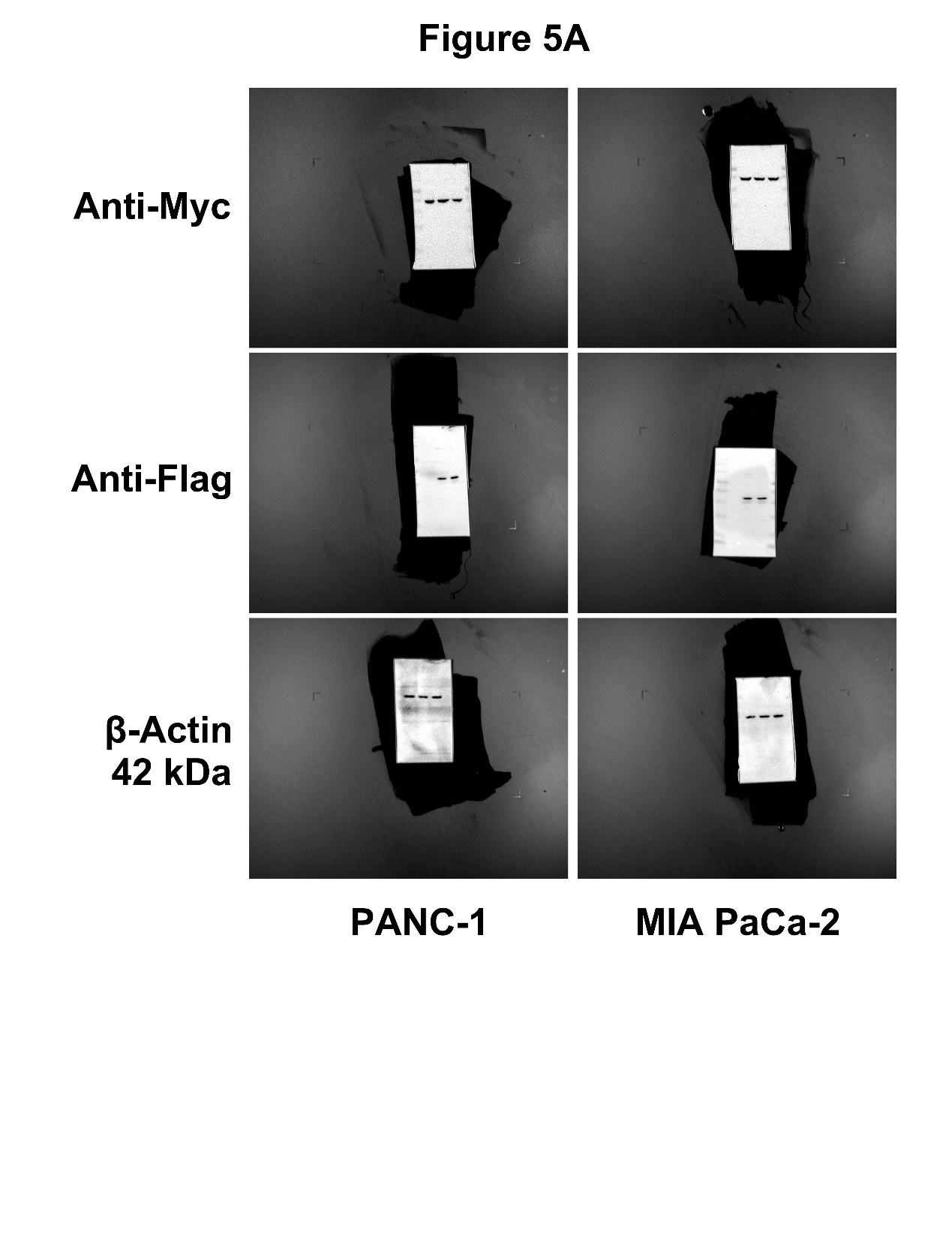


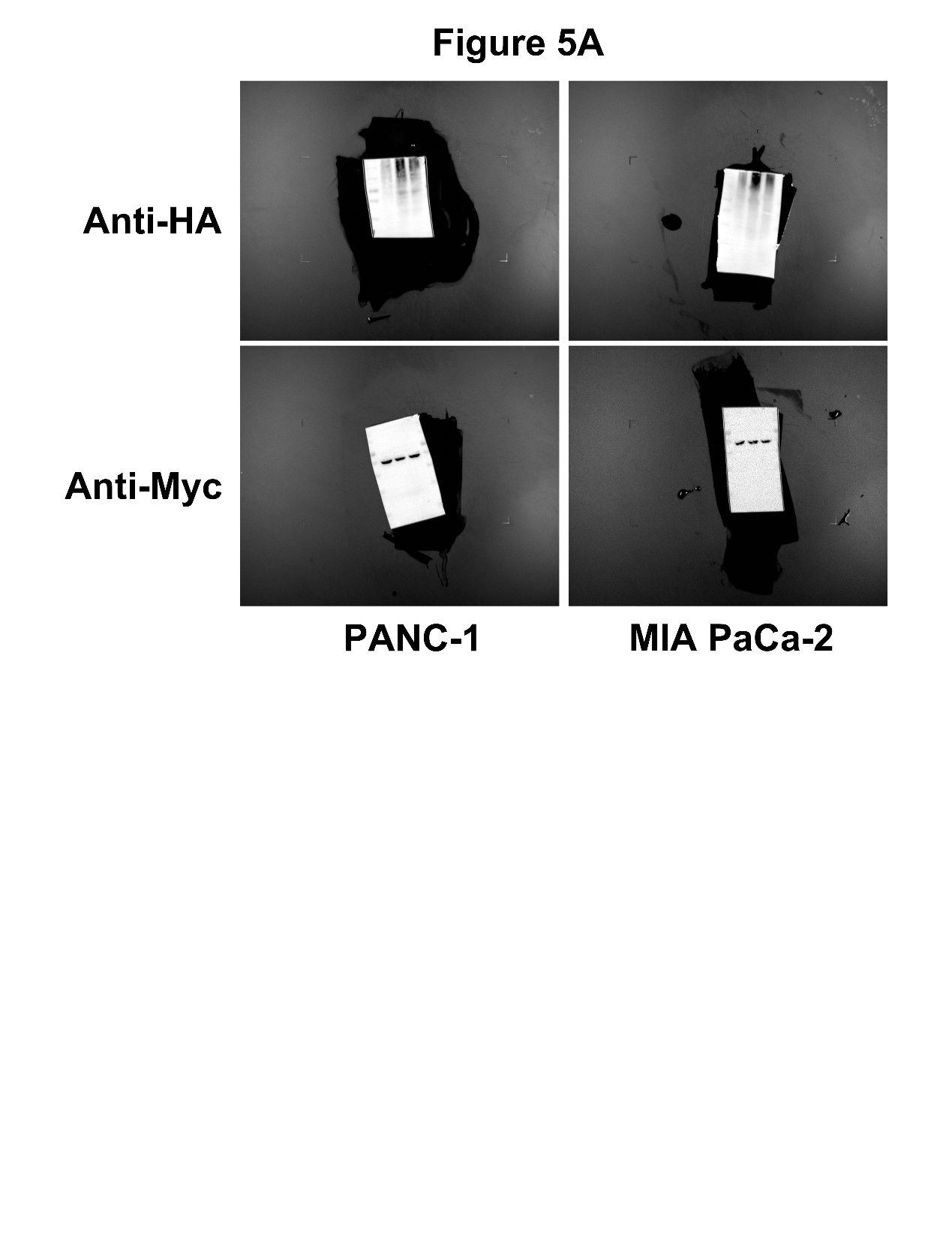


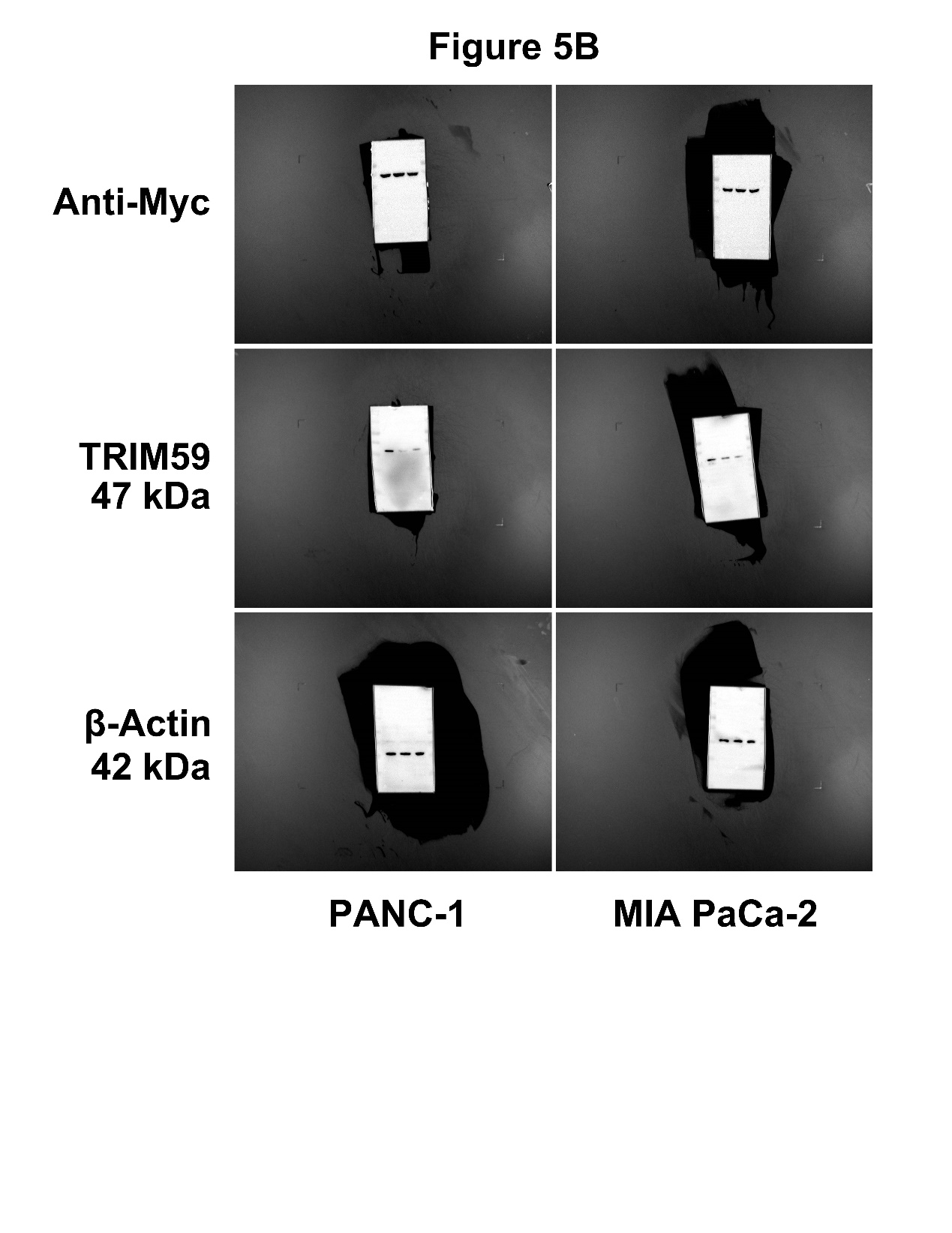


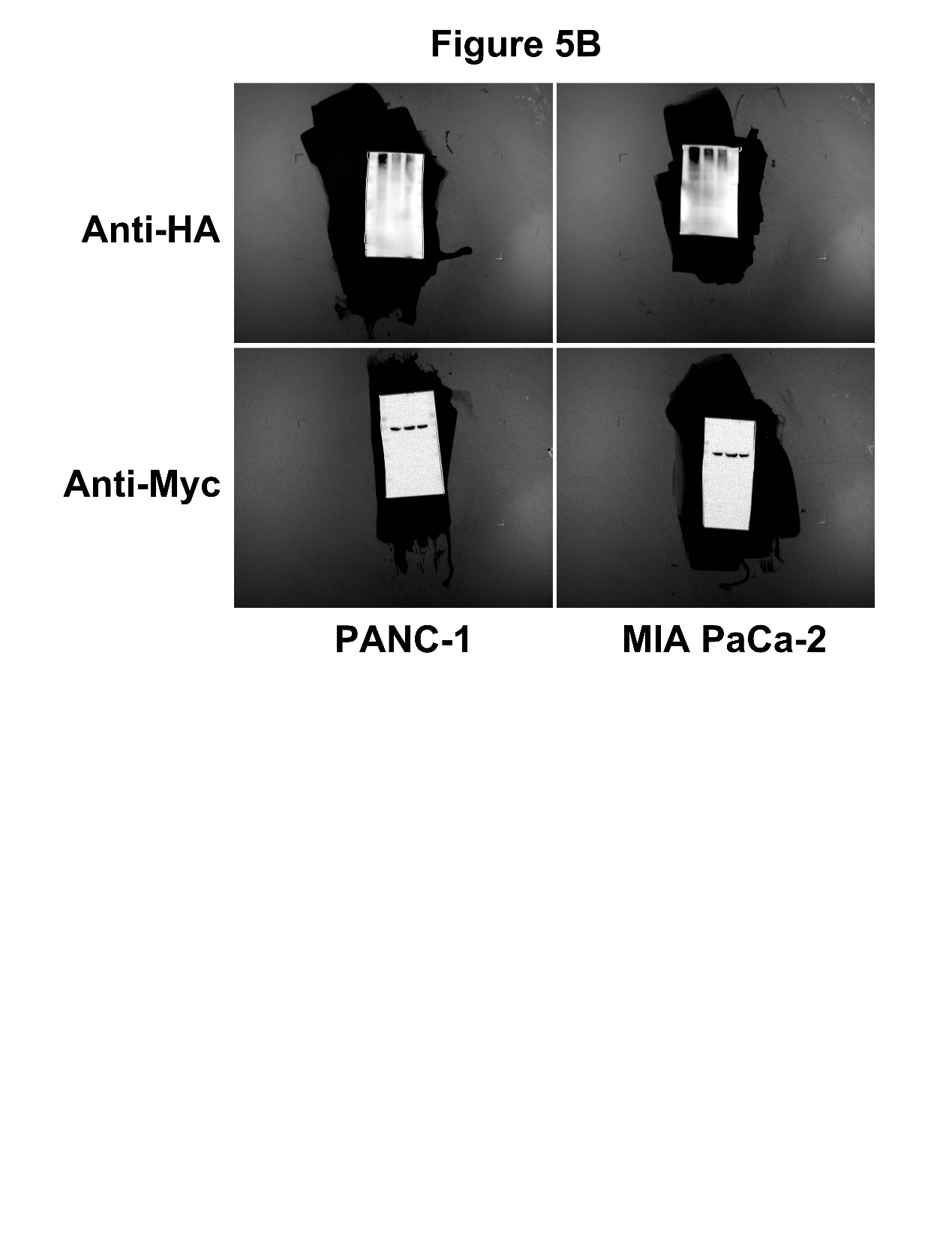


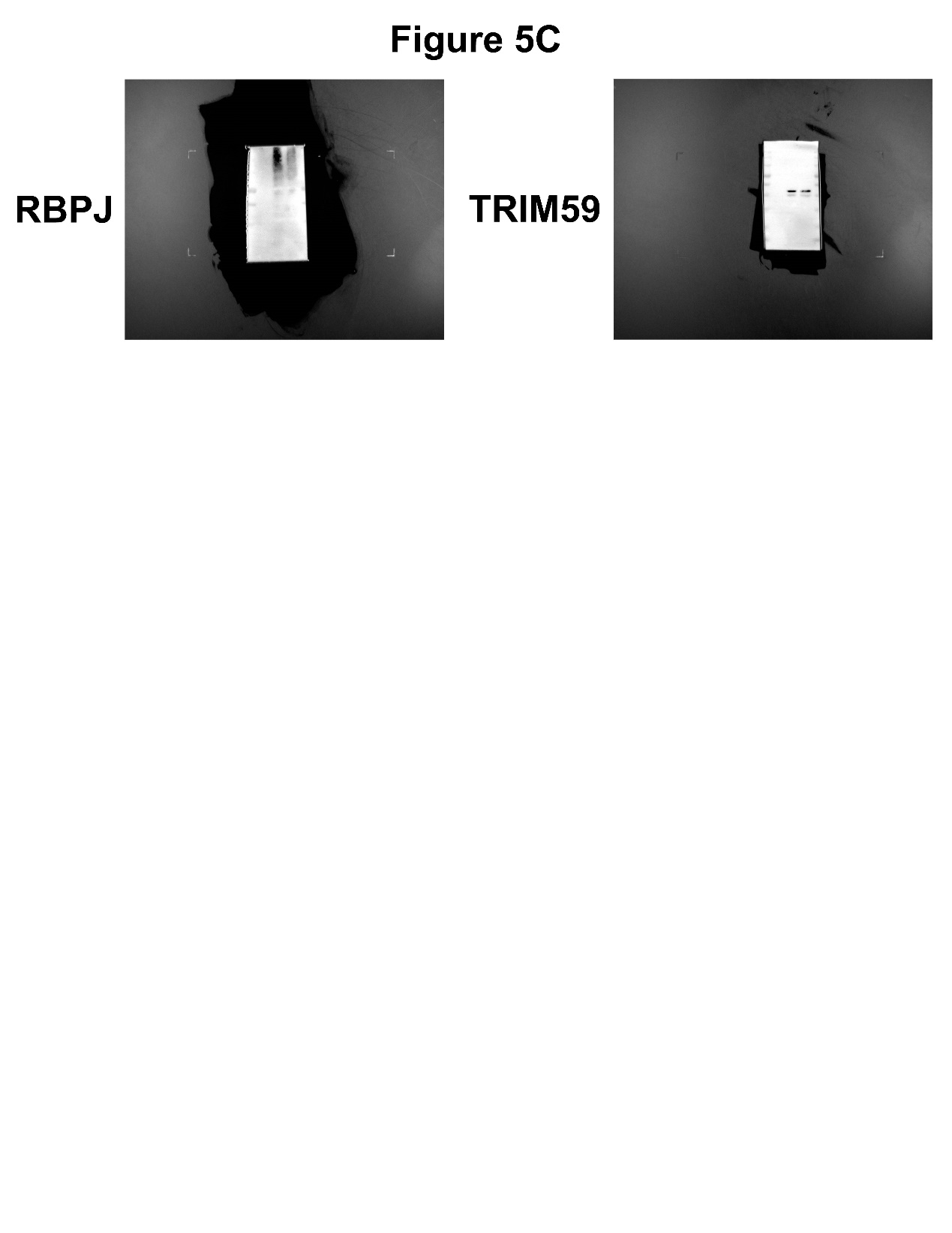


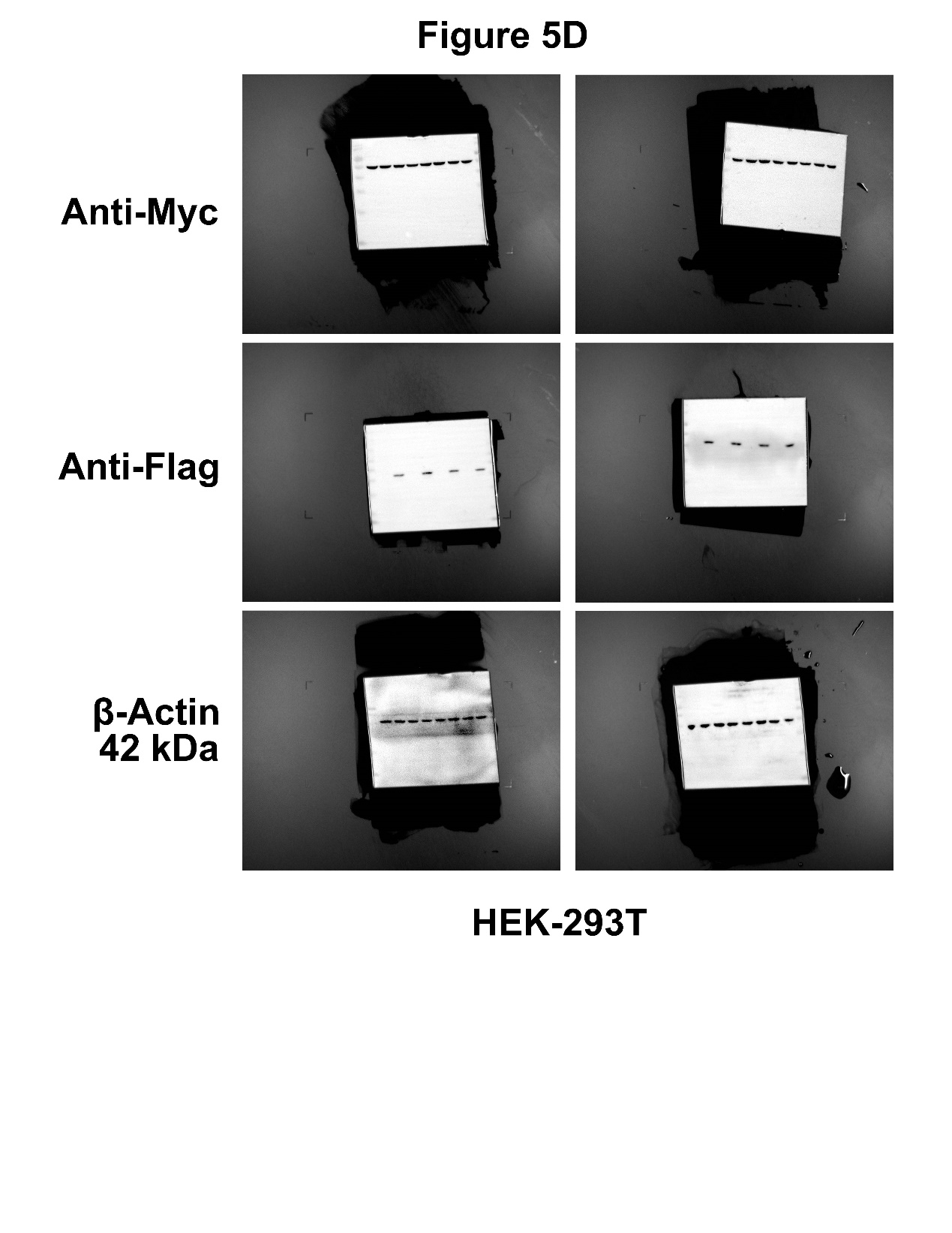


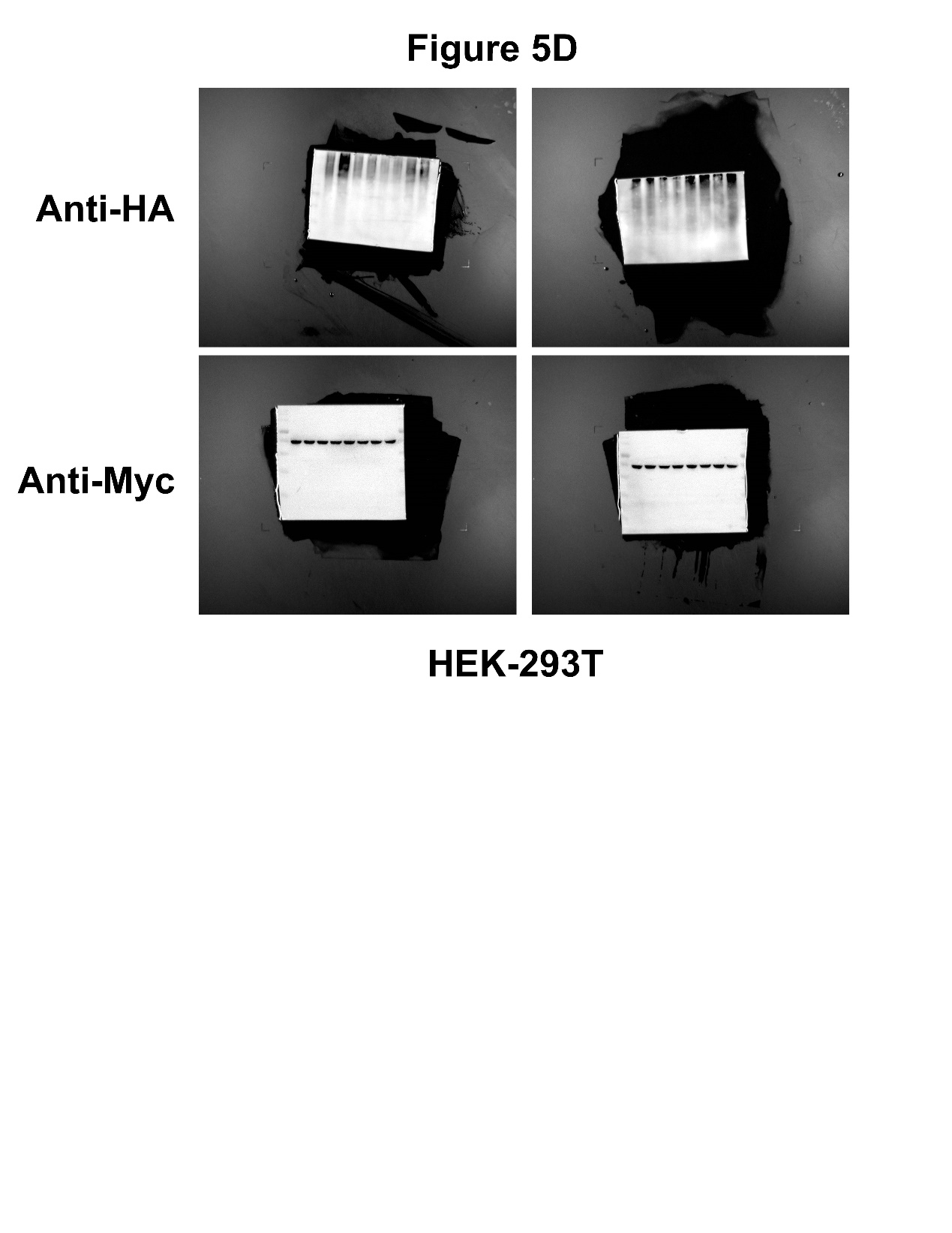


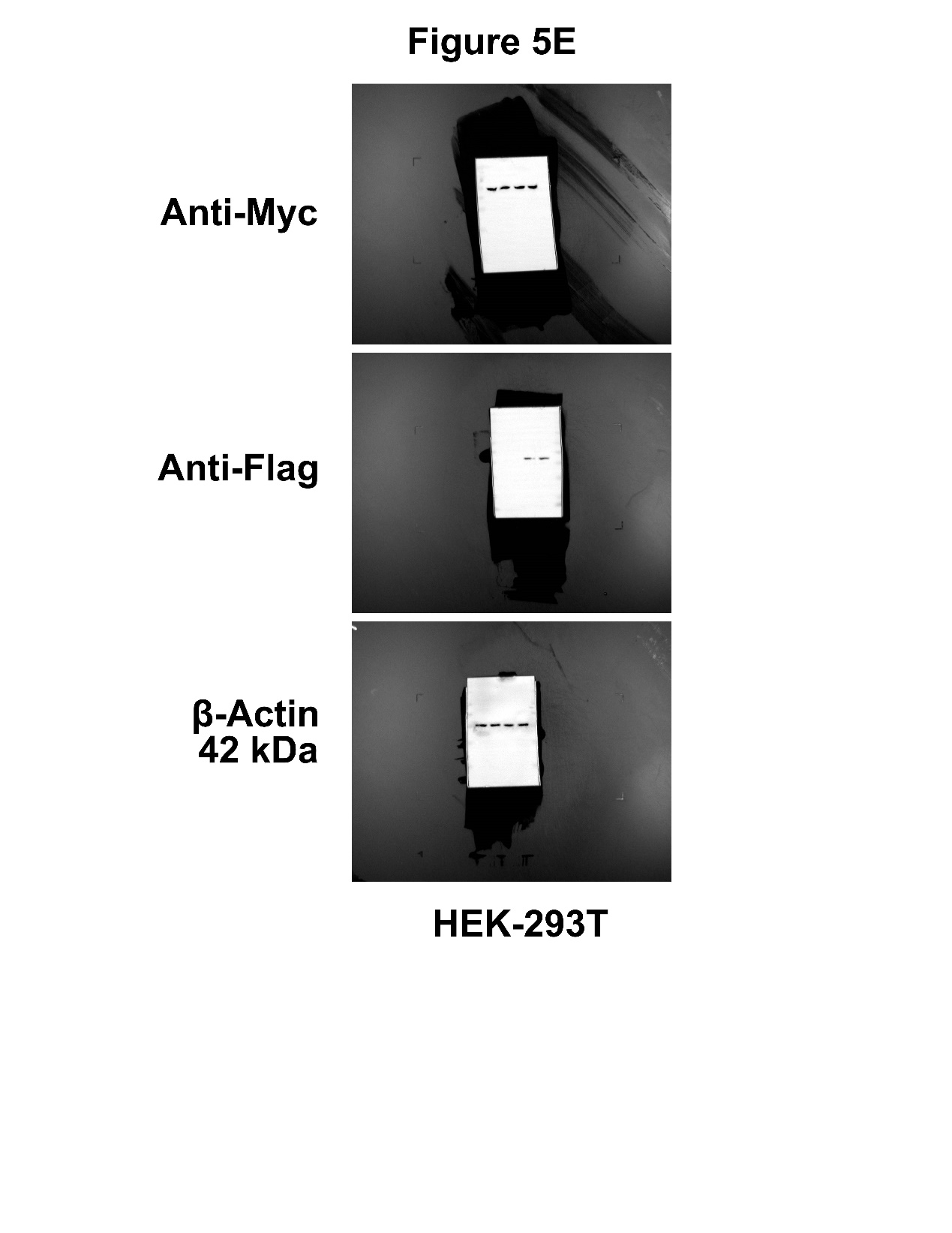


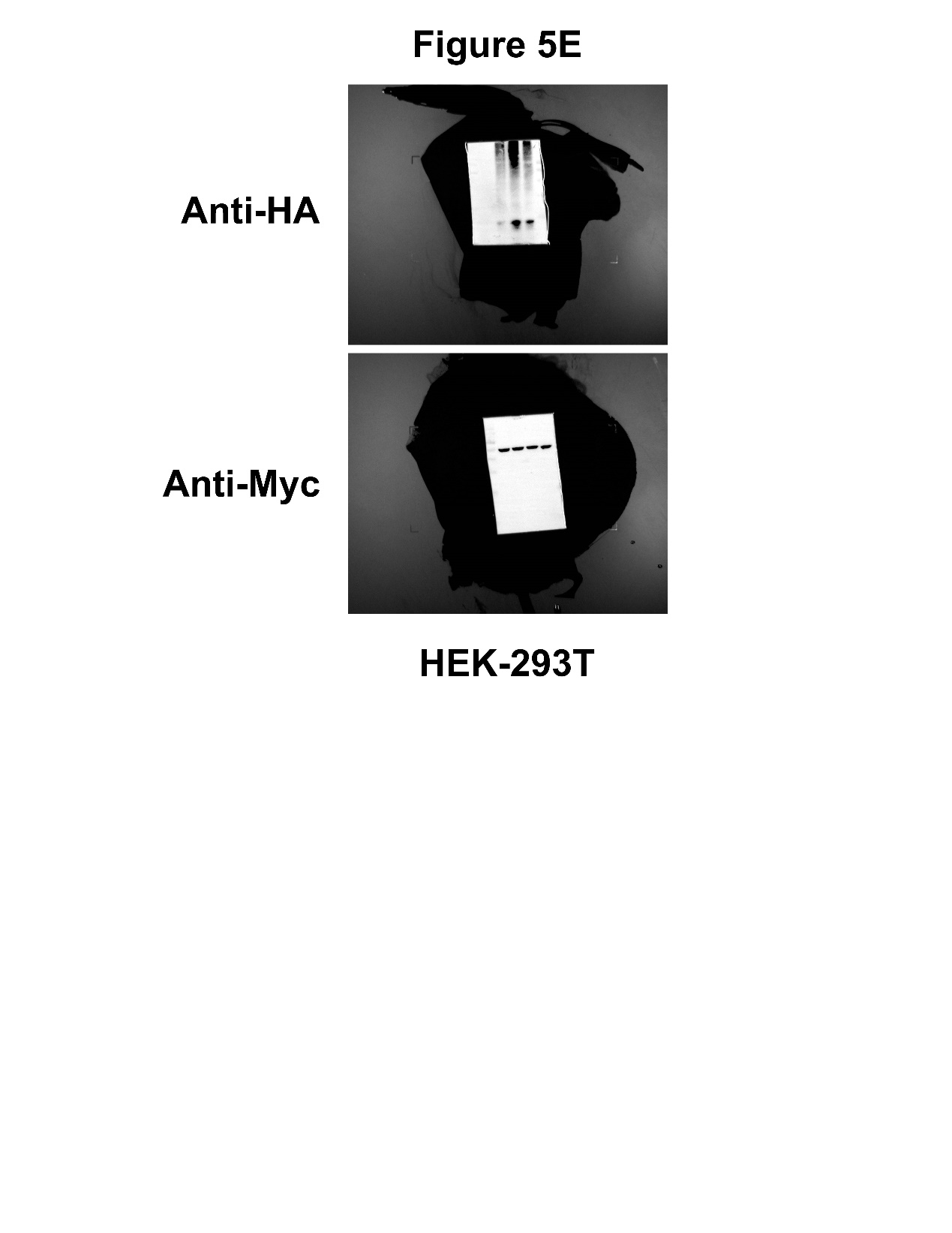


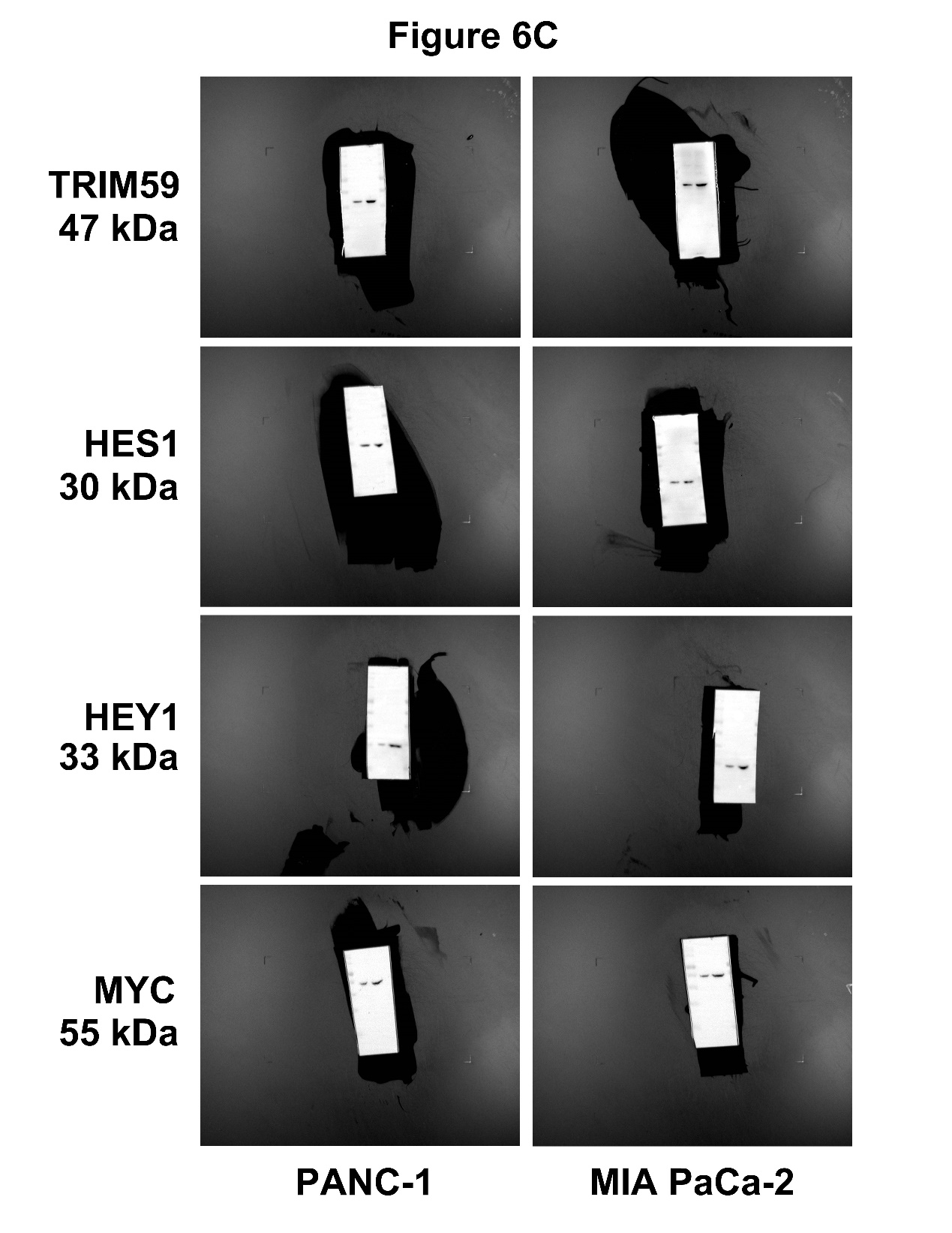


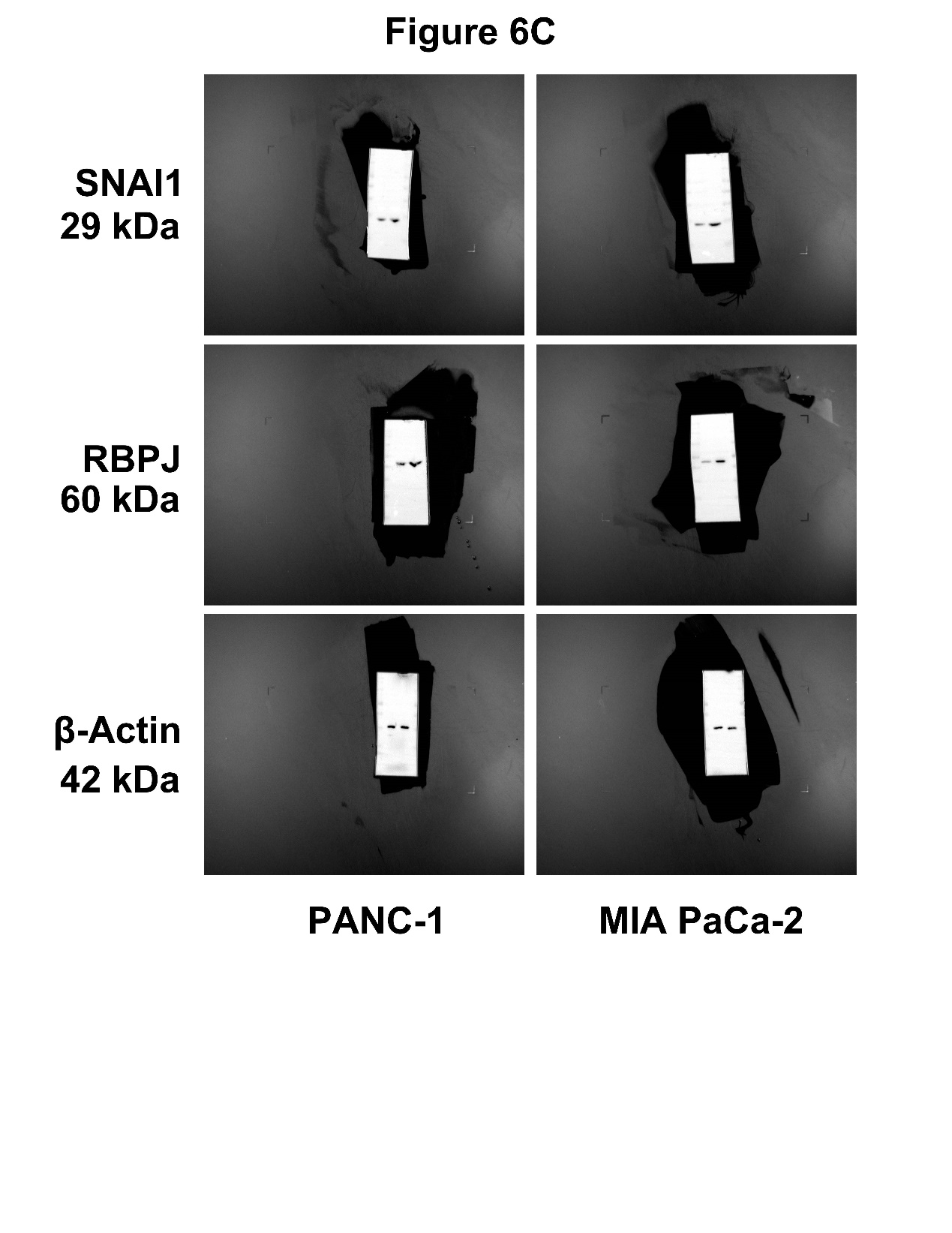


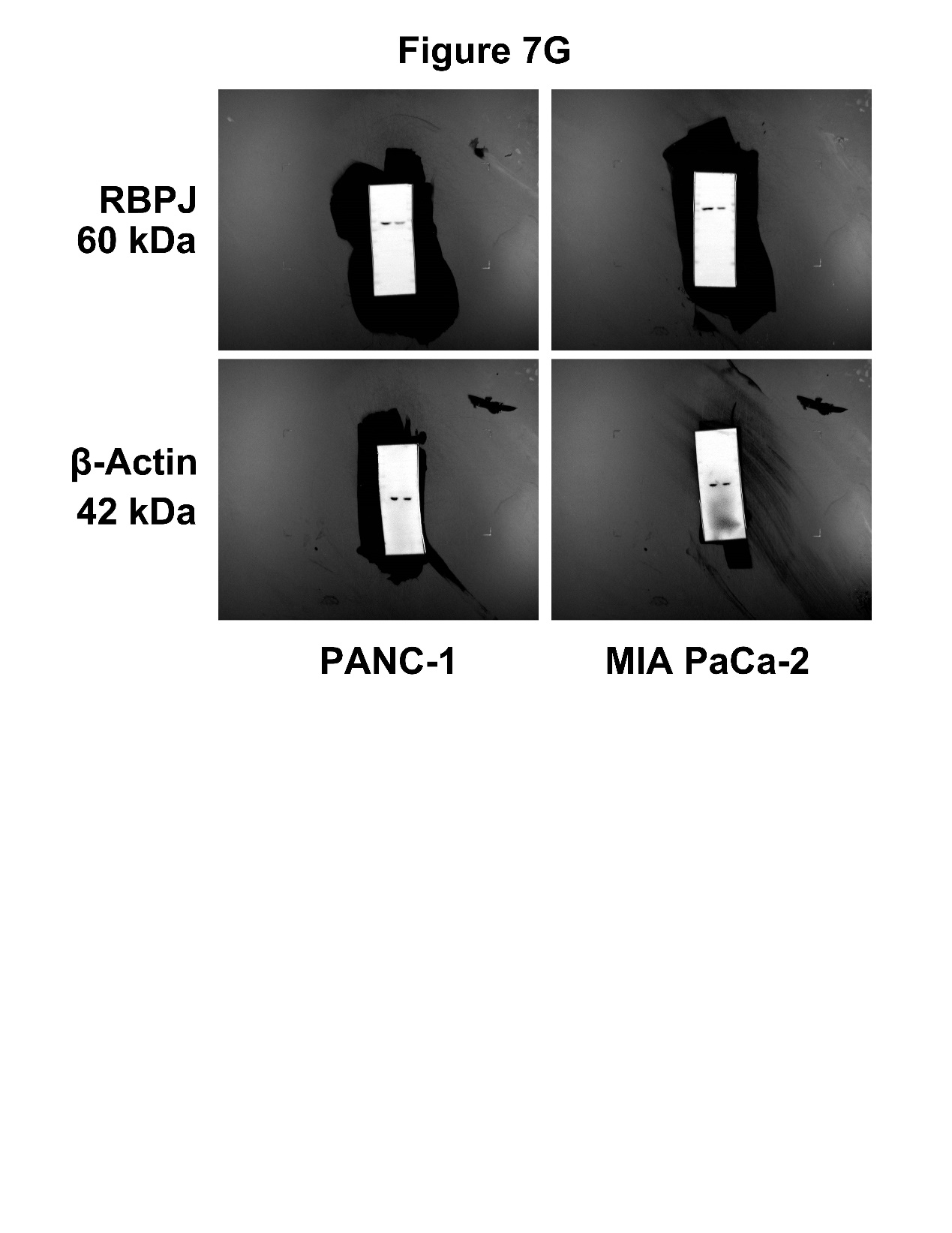


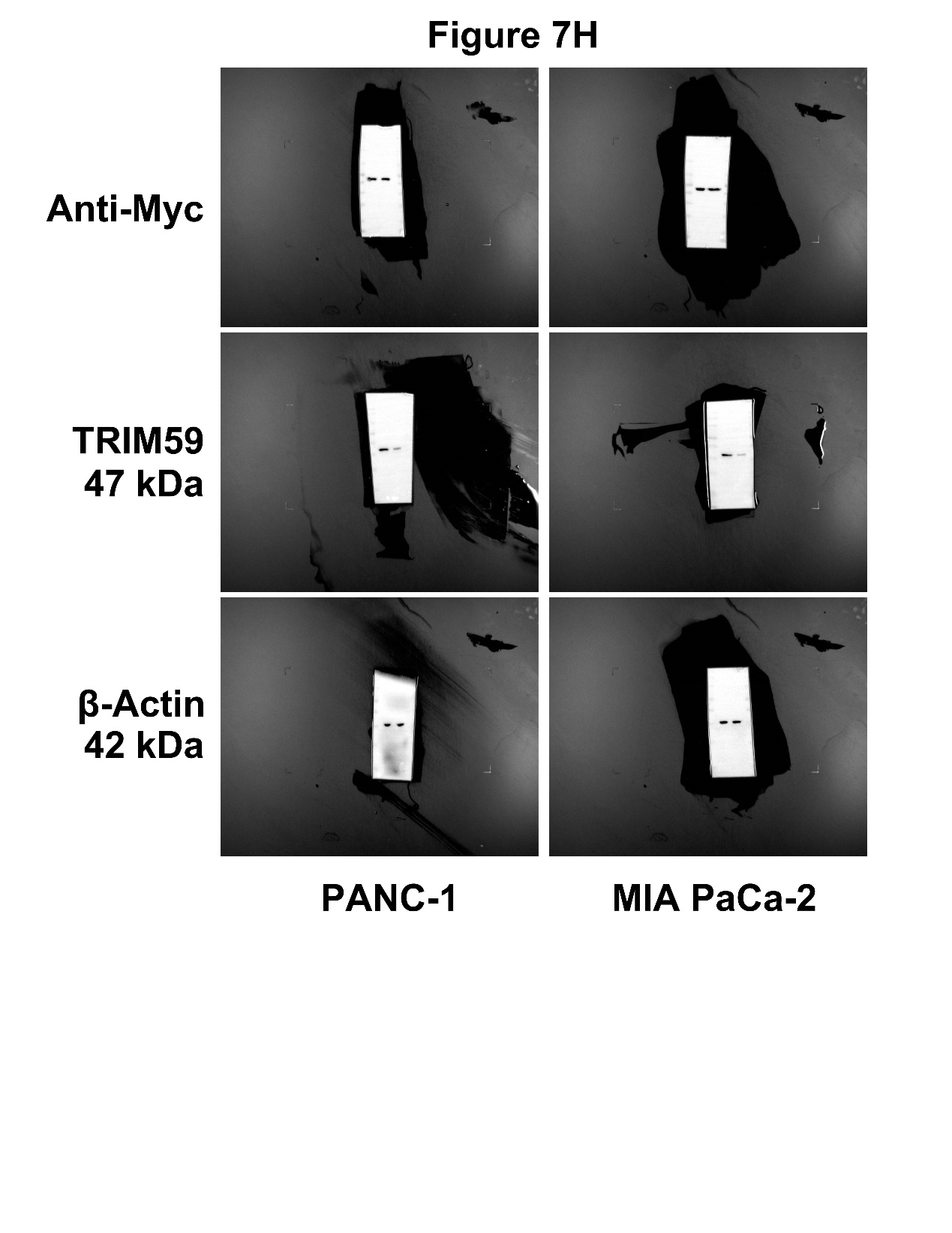


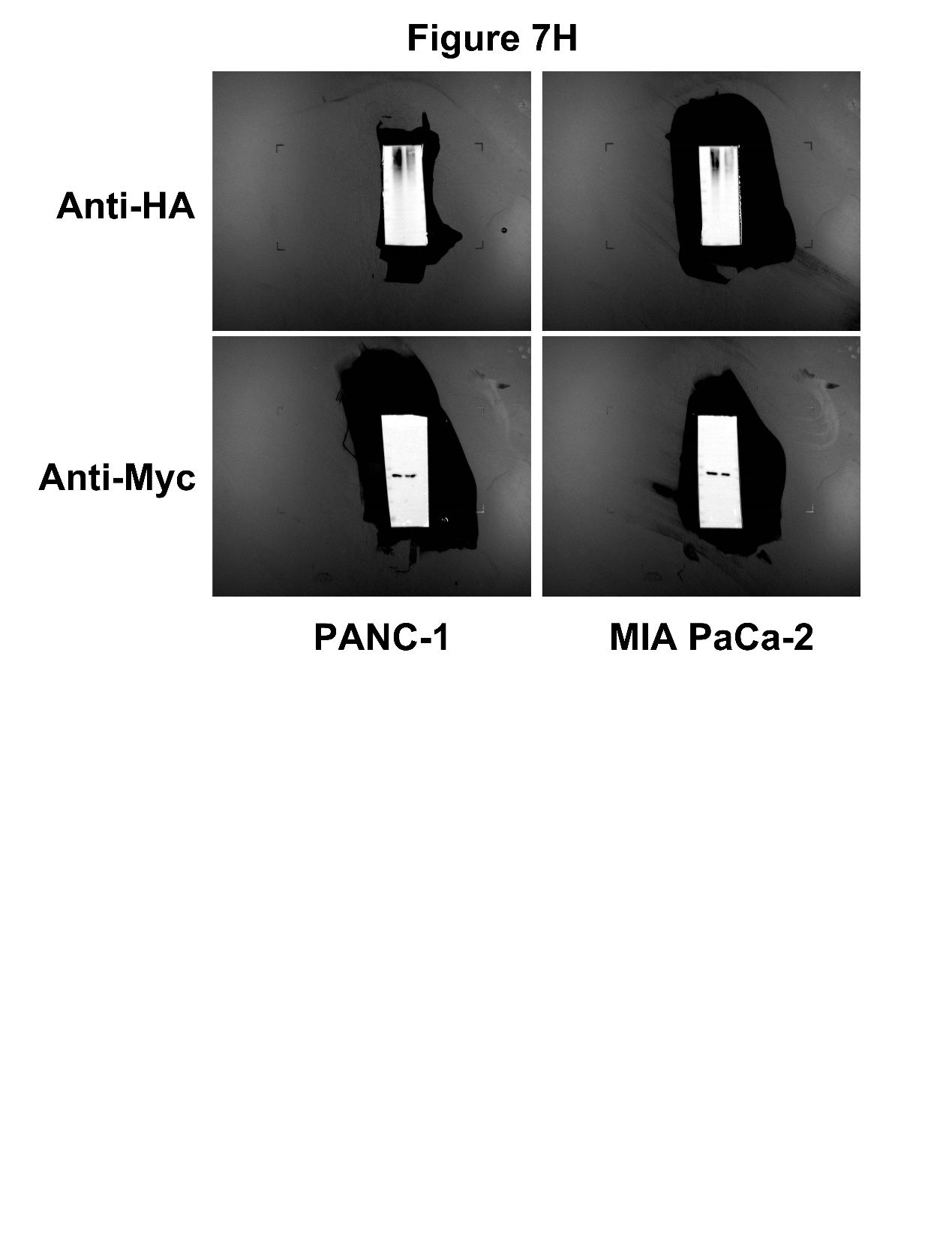

Supplement: Supplementary file 9 — Original Western Blots [file 41419_2024_7324_MOESM9_ESM.docx]
