# Supplementary material for: TRIM59/RBPJ positive feedback circuit confers gemcitabine resistance in pancreatic cancer by activating the Notch signaling pathway
Source: Cell Death Dis. 2024 Dec 26;15(12):932. doi: 10.1038/s41419-024-07324-y (PMC11671593; doi:10.1038/s41419-024-07324-y)
Supplement: Supplementary file 4 — Supplementary Figure 4 [file 41419_2024_7324_MOESM4_ESM.docx]

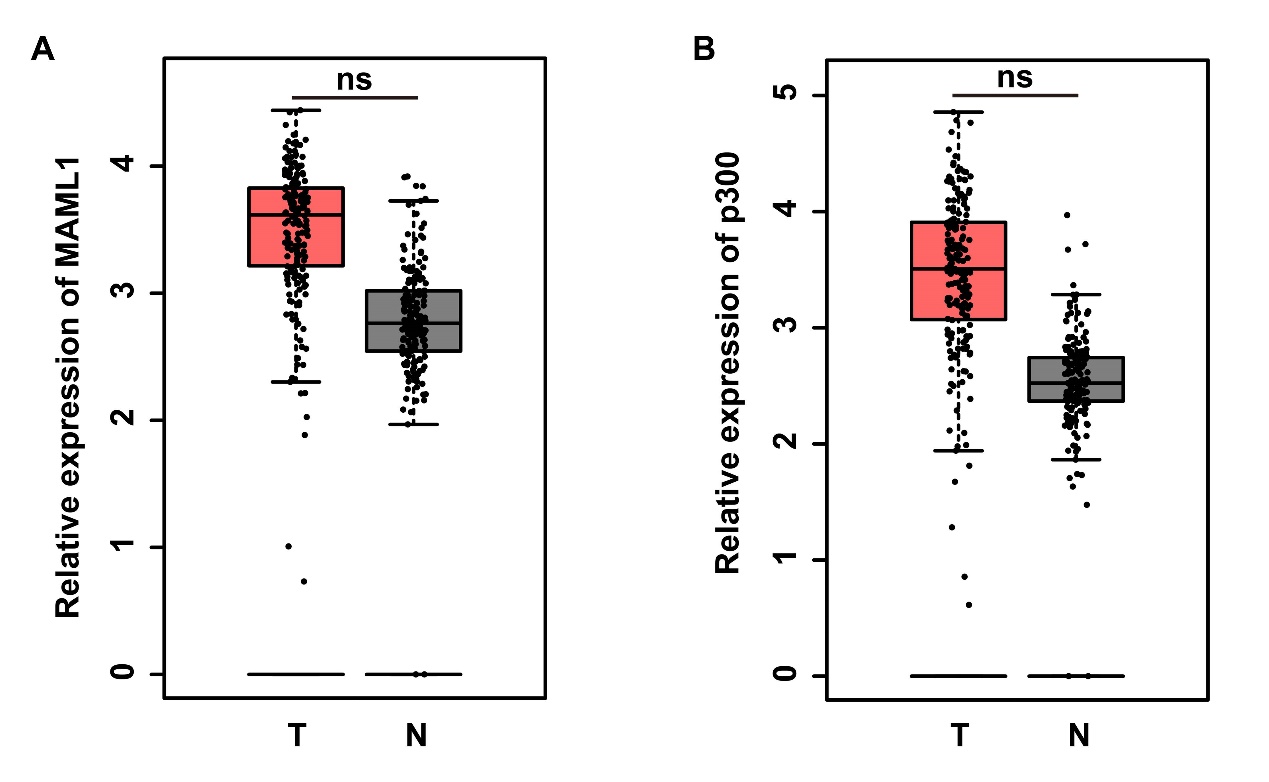


**Figure S4.** (**A, B**) The mRNA levels of MAML1 (**A**) and p300 (**B**) in the PC datasets obtained from TCGA database. ns: no significance
