# Supplementary material for: TRIM59/RBPJ positive feedback circuit confers gemcitabine resistance in pancreatic cancer by activating the Notch signaling pathway
Source: Cell Death Dis. 2024 Dec 26;15(12):932. doi: 10.1038/s41419-024-07324-y (PMC11671593; doi:10.1038/s41419-024-07324-y)
Supplement: Supplementary file 5 — Supplementary Figure 5 [file 41419_2024_7324_MOESM5_ESM.docx]

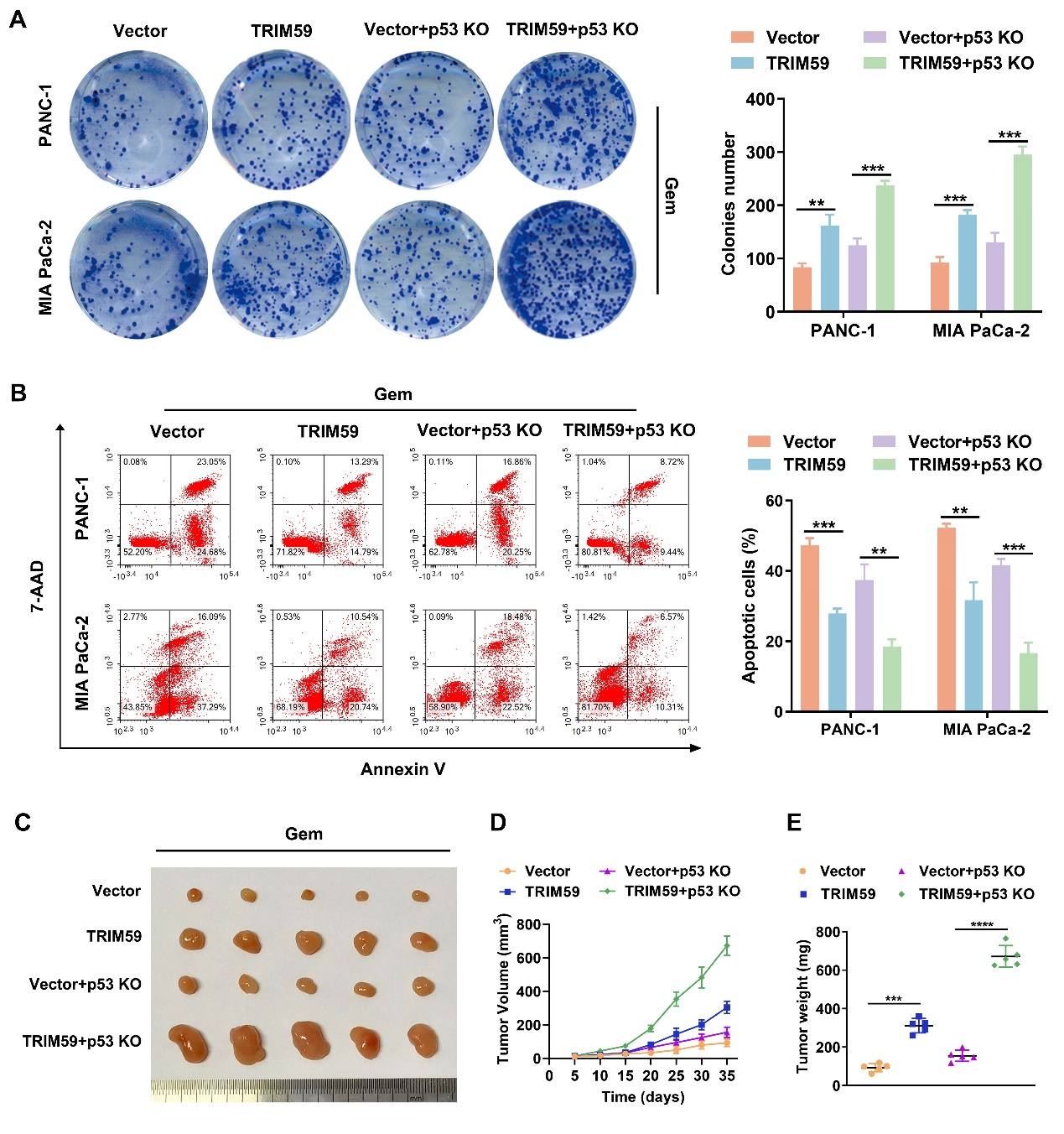


**Figure S5. The effect of TRIM59 on gemcitabine resistance of pancreatic cancer is not p53-dependent.** (**A, B**) Colony formation (**A**) and flow cytometry (**B**) analyses of the indicated PC cells treated with gemcitabine. (**C-E**) Representative images of tumor-bearing mice in the indicated PC cells after gemcitabine treatment. Tumors from all mice (**C**) and their volume (**D**), and weight (**E**) are shown. **P < 0.01, ***P < 0.001, ****P < 0.0001
