# Supplementary material for: TRIM59/RBPJ positive feedback circuit confers gemcitabine resistance in pancreatic cancer by activating the Notch signaling pathway
Source: Cell Death Dis. 2024 Dec 26;15(12):932. doi: 10.1038/s41419-024-07324-y (PMC11671593; doi:10.1038/s41419-024-07324-y)
Supplement: Supplementary file 6 — Supplementary Table 1 [file 41419_2024_7324_MOESM6_ESM.docx]

| **No.** | **Name** | **Company** | **Catalog number** | **Dilution ratio** | **Molecular weight** |
| --- | --- | --- | --- | --- | --- |
| 1 | RBPJ | Proteintech | 14613-1-AP | IP: 2 ug  WB: 1:1000 | 60 kDa |
| 2 | β-Actin | Proteintech | 66009-1-Ig | WB: 1:30000 | 42 kDa |
| 3 | HES1 | CST | #11988 | WB: 1:1000 | 30 kDa |
| 4 | HEY1 | Proteintech | 19929-1-AP | WB: 1:2000 | 33 kDa |
| 5 | MYC | Proteintech | 10828-1-AP | WB: 1:5000 | 55 kDa |
| 6 | SNAI1 | Proteintech | 13099-1-AP | WB: 1:1000 | 29 kDa |
| 7 | TRIM59 | Proteintech | 28575-1-AP | WB: 1:1000 | 47 kDa |
|  |  |  |  | IHC: 1:500  IP: 2 ug  IF: 1:200 |  |
| 8 | Myc-Tag | CST | #2276 | WB: 1:1000  IP: 1:250 |  |
| 9 | DYKDDDDK Tag (Flag-tag) | CST | #14793 | WB: 1:1000  IP: 1:50 |  |
| 10 | HA-Tag | Proteintech | 51064-2-AP | WB: 1:5000 |  |
| 11 | γ-H2A.X | CST | #9718 | IF: 1:1000 |  |
| 12 | RBPJ | Proteintech (Active Motif) | 61506 | ChIP: 1:50  IHC: 1:200  IF: 1:200 | 68 kDa |
| 13 | Ki-67 | Proteintech | 27309-1-AP | IHC: 1:2000 |  |
| 14 | Cleaved caspase 3 | Proteintech | 25128-1-AP | IHC: 1:200 |  |
| 15 | IgG (Rabbit) | Beyotime | A7016 | IP: 2 ug |  |
| 16 | IgG (Mouse) | Beyotime | A7028 | IP: 2 ug |  |
|  |  |  |  |  |  |
|  |  |  |  |  |  |
|  |  |  |  |  |  |
|  |  |  |  |  |  |

**Supplementary Table 1. Antibodies.**
