# Supplementary material for: TRIM59/RBPJ positive feedback circuit confers gemcitabine resistance in pancreatic cancer by activating the Notch signaling pathway
Source: Cell Death Dis. 2024 Dec 26;15(12):932. doi: 10.1038/s41419-024-07324-y (PMC11671593; doi:10.1038/s41419-024-07324-y)
Supplement: Supplementary file 7 — Supplementary Table 2 [file 41419_2024_7324_MOESM7_ESM.docx]

**Supplementary Table 2. RT-qPCR Primers.**

| Gene | Forward Primer (5'-3') | Reverse Primer (5'-3') |
| --- | --- | --- |
| RBPJ | AACAAATGGAACGCGATGGTT | GGCTGTGCAATAGTTCTTTCCTT |
| HES1 | CCTGTCATCCCCGTCTACAC | CACATGGAGTCCGCCGTAA |
| HEY1 | GTTCGGCTCTAGGTTCCATGT | CGTCGGCGCTTCTCAATTATTC |
| MYC | GTCAAGAGGCGAACACACAAC | TTGGACGGACAGGATGTATGC |
| SNAI1 | ACTGCAACAAGGAATACCTCAG | GCACTGGTACTTCTTGACATCTG |
| TRIM59 | AAGATCCTCGTGTACTGCCAT | CAATGCCAGTTGGAGCAATTTC |
| ACTB | CATGTACGTTGCTATCCAGGC | CTCCTTAATGTCACGCACGAT |
