# Supplementary material for: TRIM59/RBPJ positive feedback circuit confers gemcitabine resistance in pancreatic cancer by activating the Notch signaling pathway
Source: Cell Death Dis. 2024 Dec 26;15(12):932. doi: 10.1038/s41419-024-07324-y (PMC11671593; doi:10.1038/s41419-024-07324-y)
Supplement: Supplementary file 8 — Supplementary Table 3 [file 41419_2024_7324_MOESM8_ESM.docx]

**Supplementary Table 3. The relationship between TRIM59 expression and PC clinical characteristics.**

| **Clinicopathologic Feature** |  | **TRIM59** | | ***P*** |
| --- | --- | --- | --- | --- |
|  |  | **High expression** | **Low expression** |  |
| All cases |  | 50 | 48 |  |
| Age |  |  |  |  |
|  | ≤60 | 20 | 22 | 0.5597 |
|  | >60 | 30 | 26 |  |
| Gender |  |  |  |  |
|  | Male | 31 | 27 | 0.5626 |
|  | Female | 19 | 21 |  |
| T classification |  |  |  |  |
|  | T1 | 18 | 29 | **0.0156** |
|  | T2-4 | 32 | 19 |  |
| N classification |  |  |  |  |
|  | N0 | 15 | 25 | **0.0262** |
|  | N1/N2 | 35 | 23 |  |
| M classification |  |  |  |  |
|  | M0 | 47 | 46 | 0.9626 |
|  | M1 | 3 | 2 |  |
| AJCC stage |  |  |  |  |
|  | Ⅰ/Ⅱ | 39 | 40 | 0.5044 |
|  | Ⅲ/Ⅳ | 11 | 8 |  |

Bold values indicate statistically significant, P values less than 0.05.
